# The relationship between alcohol use and dementia in adults aged more than 60 years: a combined analysis of prospective, individual‐participant data from 15 international studies

**DOI:** 10.1111/add.16035

**Published:** 2022-09-04

**Authors:** Louise Mewton, Rachel Visontay, Nicholas Hoy, Darren M. Lipnicki, Matthew Sunderland, Richard B. Lipton, Maëlenn Guerchet, Karen Ritchie, Jenna Najar, Nikolaos Scarmeas, Ki‐Woong Kim, Steffi Riedel Heller, Martin van Boxtel, Erin Jacobsen, Henry Brodaty, Kaarin J. Anstey, Mary Haan, Marcia Scazufca, Elena Lobo, Perminder S. Sachdev, Collaborators from the Cohort Studies of Memory in an International Consortium (COSMIC)

**Affiliations:** ^1^ Centre for Healthy Brain Ageing University of New South Wales Sydney Australia; ^2^ Neuropsychiatric Institute The Prince of Wales Hospital Sydney Australia; ^3^ The Matilda Centre for Mental Health and Substance Use University of Sydney Sydney Australia; ^4^ Saul R. Korey, Department of Neurology, Albert Einstein College of Medicine Yeshiva University New York NY USA; ^5^ Department of Epidemiology and Population Health, Albert Einstein College of Medicine Yeshiva University New York NY USA; ^6^ Department of Psychiatry and Behavioral Medicine, Albert Einstein College of Medicine Yeshiva University New York NY USA; ^7^ Limoges University, CHU Limoges, EpiMaCT, Institute of Epidemiology and Tropical Neurology, OmegaHealth Limoges France; ^8^ Neuropsychiatry, Epidemiological and Clinical Research, La Colombière Hospital Montpellier France; ^9^ Université de Montpellier Montpellier France; ^10^ Centre for Clinical Brain Sciences University of Edinburgh Edinburgh UK; ^11^ Neuropsychiatric Epidemiology Unit, Department of Psychiatry and Neurochemistry Institute of Neuroscience and Physiology, Sahlgrenska Academy, Centre for Ageing and Health (AGECAP) at the University of Gothenburg Mölndal Sweden; ^12^ Region Västra Götaland Sahlgrenska University Hospital, Psychiatry, Cognition and Old Age Psychiatry Clinic Gothenburg Sweden; ^13^ Department of Neurology, Aiginition Hospital National and Kapodistrian University of Athens Athens Greece; ^14^ Department of Neurology Columbia University New York NY USA; ^15^ Department of Neuropsychiatry Seoul National University Bundang Hospital Seongnam South Korea; ^16^ Department of Psychiatry, College of Medicine Seoul National University Seoul South Korea; ^17^ Department of Brain and Cognitive Sciences Seoul National University Seoul South Korea; ^18^ Institute of Social Medicine, Occupational Health and Public Health (ISAP), Medical Faculty University of Leipzig Leipzig Germany; ^19^ School for Mental Health and Neuroscience/Alzheimer Centrum Limburg, Department of Psychiatry and Neuropsychology Maastricht University Maastricht the Netherlands; ^20^ Department of Psychiatry, School of Medicine University of Pittsburgh Pittsburgh PA USA; ^21^ School of Psychology University of New South Wales Sydney Australia; ^22^ Neuroscience Research Australia University of New South Wales Sydney Australia; ^23^ Centre for Research on Ageing, Health and Wellbeing Australian National University Canberra Australia; ^24^ Department of Epidemiology and Biostatistics, School of Medicine University of California San Francisco CA USA; ^25^ LIM‐23, Hospital das Clinicas (HCFMUSP), Faculdade de Medicina Universidade de São Paulo São Paulo Brazil; ^26^ Department of Preventive Medicine and Public Health Universidad de Zaragoza Zaragoza Spain; ^27^ Instituto de Investigacion Sanitaria Aragon Zaragoza Spain; ^28^ Centro de Investigacion Biomedica en Red de Salud Mental (CIBERSAM) Ministry of Science and Innovation Madrid Spain

**Keywords:** Alcohol, cross‐national comparison, dementia, epidemiology, individual participant data meta‐analysis, older adults

## Abstract

**Aim:**

To synthesize international findings on the alcohol–dementia relationship, including representation from low‐ and middle‐income countries.

**Methods:**

Individual participant data meta‐analysis of 15 prospective epidemiological cohort studies from countries situated in six continents. Cox regression investigated the dementia risk associated with alcohol use in older adults aged over 60 years. Additional analyses assessed the alcohol–dementia relationship in the sample stratified by sex and by continent. Participants included 24 478 community dwelling individuals without a history of dementia at baseline and at least one follow‐up dementia assessment. The main outcome measure was all‐cause dementia as determined by clinical interview.

**Results:**

At baseline, the mean age across studies was 71.8 (standard deviation = 7.5, range = 60–102 years), 14 260 (58.3%) were female and 13 269 (54.2%) were current drinkers. During 151 636 person‐years of follow‐up, there were 2124 incident cases of dementia (14.0 per 1000 person‐years). When compared with abstainers, the risk for dementia was lower in occasional [hazard ratio (HR) = 0.78; 95% confidence interval (CI) = 0.68–0.89], light–moderate (HR = 0.78; 95% CI = 0.70–0.87) and moderate–heavy drinkers (HR = 0.62; 95% CI = 0.51–0.77). There was no evidence of differences between life‐time abstainers and former drinkers in terms of dementia risk (HR = 0.98; 95% CI = 0.81–1.18). In dose–response analyses, moderate drinking up to 40 g/day was associated with a lower risk of dementia when compared with lif‐time abstaining. Among current drinkers, there was no consistent evidence for differences in terms of dementia risk. Results were similar when the sample was stratified by sex. When analysed at the continent level, there was considerable heterogeneity in the alcohol–dementia relationship.

**Conclusions:**

Abstinence from alcohol appears to be associated with an increased risk for all‐cause dementia. Among current drinkers, there appears to be no consistent evidence to suggest that the amount of alcohol consumed in later life is associated with dementia risk.

## INTRODUCTION

In recent decades, the estimated global prevalence of dementia has nearly tripled, from 20.2 million in 1990 to 57.4 million in 2019 [1]. By 2050, the number of individuals living with dementia globally is projected to increase to 152 million [2]. Due to increases in life expectancy and greater risk factor exposure, the largest increase in dementia prevalence is expected among those living in low‐ and middle‐income countries [2]. In the absence of disease‐modifying treatments for dementia, risk factor reduction is a fundamental strategy for preventing dementia onset [3]. To this end, in the 2020 report from the Lancet Commission for Dementia Prevention, Intervention and Care it was estimated that 40% of global dementia cases could be prevented or delayed if 12 key modifiable risk factors for dementia were eliminated [3].

Excessive or harmful alcohol use in mid‐life was newly included in the 2020 report from the Lancet Commission as one of the key modifiable risk factors for dementia [3]. This was supported by considerable evidence for the neurotoxic effects of ethanol on the brain [4, 5, 6], and by a recent study of hospital‐based records that identified alcohol use disorders as one of the strongest modifiable risk factors for dementia when compared with other established risk factors, including high blood pressure and diabetes [7]. In population‐based observational studies, often based on samples of older adults, heavy alcohol use has sometimes been found to increase the risk for dementia, although some studies have found heavy alcohol use to be unrelated to dementia risk [8]. In contrast to heavy use, population‐based studies have often found that light‐to‐moderate alcohol use appears to reduce dementia risk when compared with abstinence [8]. Overall, reviews of population‐based observational studies suggest that the alcohol–dementia relationship is likely to be J‐shaped, with low levels of alcohol use conferring some benefit when compared with abstinence from alcohol and progressively higher levels of alcohol use associated with a steadily increasing dementia risk in a dose–response trend [8, 9, 10].

While the evidence base for the alcohol–dementia relationship is large, prior meta‐analyses of published results have several limitations. There is a lack of standardization throughout studies in terms of alcohol categorization, with definitions of ‘light’, ‘moderate’ and ‘heavy’ alcohol use varying widely across studies and impeding cross‐study comparison. The abstaining group is often comprised of both former drinkers and life‐time abstainers, with former drinkers (or ‘sick quitters’) potentially driving the relationship between abstention and poorer health outcomes (i.e. reverse causation) [11]. Importantly, studies of the alcohol–dementia relationship are largely based on samples from high‐income countries [9, 10]. Evidence for the relationship between alcohol use and dementia is sparse in low‐ to middle‐income countries, where the future burden of dementia is likely to be concentrated [3], and where alcohol use is increasing [12].

The current study addresses these limitations by harmonizing individual participant‐level data from 15 prospective epidemiological cohort studies, including representation from countries situated throughout six continents, and examining the alcohol–dementia relationship. The overall aim of this study is to synthesize international findings on the alcohol–dementia relationship, including representation from low‐ and middle‐income countries.

## METHODS

### Contributing cohorts

All 15 contributing cohort studies are members of the Cohort Studies of Memory in an International Consortium (COSMIC) collaboration [13], and are detailed in Table 1. None of the cohorts reported participant exclusion criteria on the basis of alcohol use. Individuals were excluded from the current study if they were diagnosed with dementia at baseline, if they were missing baseline dementia status data, if they did not have any follow‐up dementia status assessment or if they were missing baseline alcohol use, age or sex data. For the current study, baseline year of data collection for each cohort was the first assessment occasion where both alcohol use and dementia status were assessed, and ranged from 1975 to 2011. The cohorts had various assessment schedules (two to 19 waves), follow‐up durations (4–40 years) and methods for establishing consensus diagnosis of dementia (Supporting information, Table S1). While the majority of the cohorts were based in high‐income countries, this study also includes representation from cohorts based in Brazil and the Republic of Congo. This project was approved by the University of New South Wales Human Research Ethics Committee (HC12446 and HC17292). The contributing cohort studies also had ethics approval. This study is reported as per the Strengthening the Reporting of Observational Studies in Epidemiology (STROBE) guidelines. The analysis was not pre‐registered and should therefore be considered exploratory.

**TABLE 1:** Details of contributing studies.

Study	Abbreviation	Location	Assessment years	Number of assessment waves	*n*	Criteria for dementia diagnosis
Einstein Ageing Study [14]	EAS	New York, USA	1993–2017	19	1284	DSM‐IV
Epidemiology of Dementia in Central Africa [15]	EPIDEMCA	Republic of Congo	2011–14	2	721	DSM‐IV
Etude Sante Psychologique Prevalence Risques et Traitement [16]	ESPRIT	Montpellier, France	1999–2016	7	1917	DSM‐IV
Framingham Heart Study (original cohort) [17]	FHS	Framingham, MA, USA	1975–2015	15	1658	DSM‐IV
Gothenburg H70 Birth Cohort Studies [18]	H70	Gothenburg, Sweden	2000–09	3	593	DSM‐III‐R
Hellenic Longitudinal Investigation of Ageing and Diet [19]	HELIAD	Larissa and Marousi, Greece	2009–18	2	972	DSM‐IV
Korean Longitudinal Study on Cognitive Ageing and Dementia [20]	KLOSCAD	South Korea	2009–18	4	5098	DSM‐IV
Leipzig Longitudinal Study of the Aged [21]	LEILA75+	Leipzig, Germany	1997–2014	7	851	DSM‐IV
Maastricht Ageing Study [22]	MAAS	South Limburg, the Netherlands	1993–2018	3	433	DSM‐III = R/DSM‐IV
Monongahela–Youghiogheny Healthy Ageing Team [23]	MYHAT	Small‐town region of PA, USA	2006–16	11	1652	CDR > 1
Personality and Total Health Through Life Project [24]	PATH	Canberra, Australia	2001–15	4	2238	DSM‐IV
Sacramento Area Latino Study on Ageing [25]	SALSA	Latinos living in the Sacramento area, CA, USA	1998–2008	7	1456	DSM‐IV
São Paulo Ageing and Health Study [26]	SPAH	São Paulo, Brazil	2003–08	2	1595	DSM‐IV
Sydney Memory and Ageing Study [27]	MAS	Sydney, Australia	2005–14	4	905	DSM‐IV
Zaragoza Dementia Depression Project [28]	ZARADEMP	Zaragoza, Spain	1994–2002	3	3099	DSM‐IV

CDR = Clinical Dementia Rating; DSM‐III = Diagnostic and Statistical Manual of Mental Disorders, 3rd edn; DSM‐IV = Diagnostic and Statistical Manual of Mental Disorders, 4th edn.

### Measures

Criteria for dementia diagnoses are listed in Table 1. Given variability across the contributing cohorts in terms of data collected on dementia subtypes, as well as the low population incidence of dementia, the main outcome variable for the current study was all‐cause dementia. Date of death data were also provided for 13 of the 15 cohorts included in this study, allowing the implementation of competing risks models in these datasets (date of death data were not available for the PATH and SPAH cohorts).

For each cohort, alcohol use was converted into average grams of pure ethanol per day (g/day), taking into account the type of alcoholic beverage reported (in studies where beverage type was differentiated) and the definition of a standard drink in the different national contexts. This g/day variable was used to model the dose–response relationship between alcohol use and dementia. Using data from all cohorts, a five‐level alcohol use variable was calculated that included no current alcohol use (current abstaining), occasional alcohol use (< 1.3 g/day), light–moderate alcohol use (1.3–24.9 g/day), moderate–heavy alcohol use (25–44.9 g/day) and heavy alcohol use (> 45 g/day). In 11 of the 15 cohorts, data on historical alcohol use were also available (i.e. ever consumed alcohol over the life‐time), allowing the separation of the current abstaining group into former drinkers and life‐time abstainers in these 11 cohorts. Supporting information, Table S2 includes details on assessment of alcohol use within each cohort and detailed code for processing the alcohol use data within each cohort is included in the Supporting information (pp. 31–48). Four of the 15 cohorts also included information on frequency of alcohol use among current drinkers that could be harmonized so that daily drinkers could be compared with those drinking less than daily (see Supporting information, Table S2 for details on harmonization of frequency data).

All cohorts included data on age, sex and smoking status (categorized as current, former and never smoker). Additional demographic covariates included years of education at baseline (continuous variable; data available from 14 cohorts) and body mass index at baseline (BMI; continuous variable; data available from 14 cohorts). Clinical covariates included baseline depression status (absent/present; data available from all cohorts), a history of stroke at baseline (absent/present; data available from 14 cohorts), a history of diabetes at baseline (absent/present; data available from all cohorts), a history of myocardial infarction at baseline (absent/present; data available from 13 cohorts), hypertension at baseline (absent/present; data available from all cohorts) and high cholesterol at baseline (absent/present; data available from 14 cohorts). Supporting information, Tables S3–S8 include detail on the assessment, harmonization and distribution of all demographic and clinical covariates.

### Statistical analysis

The proportion of missing data was generally less than 5% for any given covariate within a cohort, although extensive missing data were present for some covariates in some cohorts (see Supporting information, Tables S7 and S8 for details on missing data on baseline covariates). Prior to analysis, multiple imputation was used to account for missing data on baseline covariates within each cohort. For each cohort, 20 imputed data sets were created using the *mice* package in R [29]. To correct for the presence of dependent censoring, inverse probability of censoring weights were calculated using the *WeightIt* package in R [30]. See Supporting information for further details on multiple imputation and weight generation, as well as the R code. Data from individual cohorts were combined and analysed using a one‐stage individual participant data meta‐analytical approach. Event times were censored at the end of follow‐up/participant drop‐out, date of dementia diagnosis or date of death. A *P*‐value of 0.05 was considered statistically significant, and 95% confidence intervals (CI) are reported.

#### Alcohol use categories

Analyses first focused on the categorical alcohol use variable and were conducted in the full sample with current abstainers as the reference category. All analyses were then repeated in the sample of 11 cohorts where life‐time abstainers and former drinkers could be separated, with life‐time abstainers as the reference category. These analyses included inverse probability of censoring weights and were adjusted for age, sex and smoking status, as well as a random effect for cohort using the *coxme* package in R [31]. To identify sex‐specific relationships between alcohol use and dementia, these analyses were repeated in males and females.

Next, analyses were repeated in the subsample of cohorts that allowed adjustment for all additional demographic and clinical covariates considered (i.e. education, BMI, depression, stroke, diabetes, myocardial infarction, hypertension and high cholesterol). These ‘fully adjusted’ analyses were conducted to determine whether the relationship between dementia and alcohol use was robust to potential confounders.

Analyses were then conducted that accounted for competing risks of mortality in the cohorts that provided date of death data. This analysis accounted for the possibility that those who died may have developed dementia in the future. Competing risks models were conducted using the *survival* package in R [32], adjusting for age, sex and smoking status, as well as cohort as a clustering variable (as opposed to a random effect). All subgroup analyses based on sex, covariate adjustment and competing risks were planned a priori.

#### Dose–response curves

Dose–response analyses were first conducted with 0 g/day as the reference value. Former drinkers were excluded from these analyses and were therefore conducted in 11 of the 15 cohorts where life‐time abstainers could be separated from former drinkers. To allow health guidance among drinkers, these analyses were repeated using current drinkers only from each of the 15 cohorts, with the lowest volume of alcohol consumed per day set as the reference value (0.3 g/day). The *rms* package [33] in R was used to calculate hazard ratios for alcohol use modelled using restricted cubic splines (three knots at the 10th, 50th and 90th percentiles). These models included inverse probability of censoring weights and adjusted for the fixed effects of age, sex and smoking status, as well as cohort as a cluster variable. These analyses were also repeated in subsamples of males and females and in the subsample of cohorts that allowed for adjustment of all demographic and clinical covariates considered.

#### *Post‐hoc* sensitivity analyses

To correct for measurement error and within‐person variability in alcohol use over time, multi‐level regression calibration was implemented using information from 66 898 follow‐up assessments in 15 433 participants from 12 cohorts. A regression dilution ratio was estimated from a calibration model that regressed follow‐up alcohol consumption measurements on baseline alcohol consumption, adjusted for duration of follow‐up and baseline age, sex, smoking status, education, BMI, depression, stroke, diabetes, hypertension and high cholesterol. Nested random effects for follow‐up wave and study were also included in the calibration model. The resulting regression dilution ratio of 0.46 was extracted from this calibration model and hazard ratios (HRs) and confidence intervals were divided by this estimate to derive dose–response curves that accounted for measurement error and within‐person variability in alcohol use over time [34, 35, 36].

*Post‐hoc* sensitivity analyses were also conducted to determine whether the results replicated within cohorts grouped by continent where there were sufficient data, as well as to determine whether the results replicated after those reporting stroke at baseline were excluded from the analysis.

The relationship between daily drinking and dementia risk was also examined in four cohorts that included consistent information on frequency of alcohol use. These analyses were conducted with the *coxme* package in R and included the binary drinking frequency variable (daily drinking/not daily drinking) while adjusting for age, sex, smoking status and baseline alcohol consumption (g/day), as well as cohort as a random effect.

## RESULTS

The combined sample of 15 cohorts included 33 532 individuals. Of these, 1522 individuals were excluded from the current study due to a dementia diagnosis at baseline, 270 had missing baseline dementia status data, 832 did not have baseline alcohol use data, six did not have data on sex and 6424 did not have any follow‐up dementia status assessment. The final analytical sample consisted of 24 478 individuals. Those included and excluded from the analyses differed in terms of alcohol use, as well as demographic and clinical characteristics (see Supporting information, Tables S9 and S10).

At baseline, the mean age across studies was 71.8 [standard deviation (SD) = 7.5, range = 60–102 years], 14 260 (58.3%) were female and 13 269 (54.2%) were current drinkers (Table 2). During 151 636 person‐years of follow‐up, there were 2124 incident cases of dementia (14.0 per 1000 person‐years). Baseline drinking patterns varied considerably across cohorts, particularly with respect to the number of abstainers (Table 2), as did demographic and clinical characteristics (Table 2, Supporting information, Tables S7 and S8). The relationships between each of the demographic and clinical characteristics and dementia risk in the combined sample are reported in Supporting information, Table S11.

**TABLE 2:** Alcohol use and basic demographic characteristics of contributing cohorts.

	Current drinker categories for individual participant data meta‐analysis						Age	Sex (female)	Smoking status		
	Abstainer^a^ % (*n*)	Former drinker % (*n*)	< 1.3 g/day % (*n*)	1.3–24.9 g/day % (*n*)	25–44.9 g/day % (*n*)	≥ 45 g/day % (*n*)	Mean (SD)	% (*n*)	Current % (*n*)	Former % (*n*)	Never % (*n*)
EAS (1284)	28.4 (365)	18.2 (234)	29.4 (378)	36.3 (466)	5.3 (68)	0.5 (7)	78.0 (5.4)	61.0 (783)	6.8 (87)	46.8 (601)	45.2 (581)
EPIDEMCA (721)	68.7 (495)	–	10.7 (77)	16.8 (121)	2.5 (18)	1.4 (10)	73.6 (6.6)	58.4 (421)	13.3 (96)	5.8 (42)	80.6 (581)
ESPRIT (1917)	16.6 (318)	3.2 (61)	8.4 (161)	56.2 (1078)	13.1 (251)	5.7 (109)	72.9 (5.4)	59.0 (1130)	6.4 (123)	35.6 (681)	58.0 (1112)
FHS (1658)	36.9 (612)	–	11.3 (187)	34.4 (571)	11.9 (198)	5.4 (90)	71.0 (6.5)	60.6 (1005)	16.3 (271)	31.8 (527)	50.1 (831)
H70 (576)	12.7 (73)	4.5 (26)	–	81.9 (472)	3.6 (21)	1.7 (10)	73.4 (4.9)	73.3 (422)	10.4 (60)	28.8 (166)	40.8 (235)
HELIAD (954)	58.6 (559)	16.0 (148)	6.1 (59)	29.4 (280)	3.8 (36)	2.1 (20)	72.4 (5.3)	60.7 (579)	10.2 (97)	26.8 (256)	62.8 (599)
KLOSCAD (5139)	67.6 (3475)	7.8 (398)	4.8 (246)	18.9 (972)	4.3 (220)	4.4 (226)	69.7 (6.4)	56.7 (2915)	8.2 (422)	21.7 (1113)	69.8 (3589)
LEILA75+ (851)	12.0 (102)	9.4 (80)	41.4 (352)	39.1 (333)	6.0 (51)	1.5 (13)	81.2 (4.8)	74.2 (632)	1.8 (15)	24.7 (210)	68.3 (581)
MAAS (433)	23.8 (103)	–	19.9 (86)	51.3 (222)	0.5 (2)	4.6 (20)	68.4 (5.7)	50.6 (219)	19.6 (85)	43.6 (189)	36.7 (159)
MYHAT (1652)	33.5 (554)	19.5 (322)	36.2 (598)	25.9 (428)	3.8 (62)	0.6 (10)	77.4 (7.3)	62.2 (1028)	6.9 (114)	45.2 (746)	47.7 (788)
PATH (2238)	13.3 (299)	8.4 (189)	15.5 (348)	56.9 (1274)	13.3 (297)	0.9 (20)	62.5 (1.5)	48.4 (1084)	9.7 (218)	37.2 (833)	53.0 (1187)
SALSA (1456)	43.6 (635)	–	31.9 (465)	17.5 (255)	4.1 (59)	2.9 (42)	70.2 (6.6)	58.3 (849)	11.0 (160)	43.3 (630)	45.7 (666)
SPAH (1595)	80.7 (1287)	23.9 (381)	–	12.2 (194)	1.1 (18)	6.0 (96)	71.6 (5.7)	61.6 (983)	13.2 (211)	40.9 (653)	45.8 (731)
Sydney MAS (905)	12.3 (111)	6.2 (56)	25.3 (229)	37.8 (342)	16.0 (145)	8.6 (78)	78.6 (4.8)	54.4 (492)	3.0 (27)	50.6 (458)	46.2 (418)
Zarademp (3099)	71.7 (2221)	11.2 (347)	2.2 (69)	19.9 (618)	4.4 (135)	1.8 (56)	72.0 (8.5)	55.4 (1718)	13.6 (420)	22.0 (683)	64.4 (1996)
Total (24478)	45.8 (11209)	16.7 (4091)	13.3 (3255)	31.2 (7626)	6.5 (1581)	3.3 (807)	71.8 (7.5)	58.3 (14260)	10.0 (2452)	31.8 (7788)	57.2 (14008)

EAS = Einstein Aging Study; EPIDEMCA = Epidemiology of Dementia in Central Africa; ESPRIT = Etude Santé Psychologique Prévalence Risques et Traitement; FHS = Framingham Heart Study; H70 : Gothenburg H70 Birth Cohort Studies; HELIAD = Hellenic Longitudinal Investigation of Aging and Diet; KLOSCAD = Korean Longitudinal Study on Cognitive Aging and Dementia; LEILA75+ = Leipzig Longitudinal Study of the Aged; MAAS = Maastricht Ageing Study; MYHAT = Monongahela–Youghiogheny Healthy Aging Team; PATH = Personality and Total Health Through Life Project; SALSA = Sacramento Area Latino Study on Aging; SPAH = São Paulo Ageing and Health Study; Sydney MAS = Sydney Memory and Ageing Study; ZARADEMP = Zaragoza Dementia Depression Project; SD = standard deviation.

^a^ Abstainer group includes both life‐time abstainers and former drinkers.

### Alcohol use categories

When compared with abstainers, the risk for dementia was lower in occasional, light–moderate and moderate–heavy drinkers (Table 3). Similar relationships were found in the fully adjusted model and competing risk model, as well as in the subsample of males. For women, the unadjusted model showed that, when compared with abstainers, the risk for dementia was lower in occasional, light–moderate and moderate–heavy drinkers. There was no evidence of a relationship between alcohol use and dementia in females when the models were fully adjusted and when the model adjusted for competing risk of death.

**TABLE 3:** Combined sample hazard ratios.

	Main model^a^	Fully adjusted model^b^	Competing risk model^c^
Combined sample	*n* = 24 478	*n* = 20 878	*n* = 20 645
Abstainer	Ref	Ref	Ref
≤ 1.3 g/day	**0.78 (0.68, 0.89)**	**0.82 (0.71, 0.96)**	**0.73 (0.54, 0.97)**
1.3–24.9 g/day	**0.78 (0.70, 0.87)**	**0.85 (0.76, 0.96)**	**0.78 (0.64, 0.95)**
25–44.9 g/day	**0.62 (0.51, 0.75)**	**0.74 (0.60, 0.90)**	**0.65 (0.45, 0.93)**
≥ 45 g/day	0.81 (0.61, 1.08)	0.78 (0.57, 1.05)	0.79 (0.58, 1.06)
Male sample	*n* = 10 218	*n* = 8873	*n* = 8452
Abstainer	Ref	Ref	Ref
≤ 1.3 g/day	**0.69 (0.53, 0.91)**	**0.71 (0.53, 0.95)**	**0.62 (0.46, 0.82)**
1.3–24.9 g/day	**0.74 (0.62, 0.88)**	**0.84 (0.69, 1.02)**	**0.70 (0.55, 0.89)**
25–44.9 g/day	**0.59 (0.46, 0.77)**	**0.70 (0.54, 0.92)**	**0.61 (0.44, 0.84)**
≥ 45 g/day	0.82 (0.60, 1.13)	0.80 (0.57, 1.13)	0.78 (0.58, 1.04)
Female sample	*n* = 14 260	*n* = 12 005	*n* = 12 193
Abstainer	Ref	Ref	Ref
≤ 1.3 g/day	**0.80 (0.68, 0.94)**	0.88 (0.73, 1.05)	0.76 (0.53, 1.09)
1.3–24.9 g/day	**0.82 (0.71, 0.94)**	0.88 (0.76, 1.02)	0.82 (0.67, 1.01)
25–44.9 g/day	**0.63 (0.46, 0.88)**	0.76 (0.55, 1.06)	0.66 (0.40, 1.10)
≥ 45 g/day	0.37 (0.12, 1.11)	0.36 (0.12, 1.10)	0.40 (0.12, 1.35)

*Note*: Bold = statistically significant at *p* < .05.

^a^ Model included all 15 cohorts and adjusted for age, sex, smoking status and random effect of study;

^b^ Model included 11 cohorts and adjusted for age, sex, smoking status, education, body mass index (BMI), depression, stroke, diabetes, myocardial infarction, hypertension, high cholesterol and random effect for study;

^c^ Model included 13 cohorts, adjusted for age, sex, smoking status and study as a cluster variable and accounted for competing risk of death.

In the 11 cohorts where life‐time abstainers could be separated from former drinkers (*n* = 20 187; Supporting information, Table S12), there was no evidence for statistically significant differences between life‐time abstainers (reference group) and former drinkers in terms of dementia risk. There was similarly no evidence for statistically significant differences between life‐time abstainers and former drinkers in the subsample of males, the subsample of females, when the analyses were fully adjusted for all demographic and clinical characteristics and when the competing risk of death was taken into account.

### Dose–response curves

**FIGURE 1:**
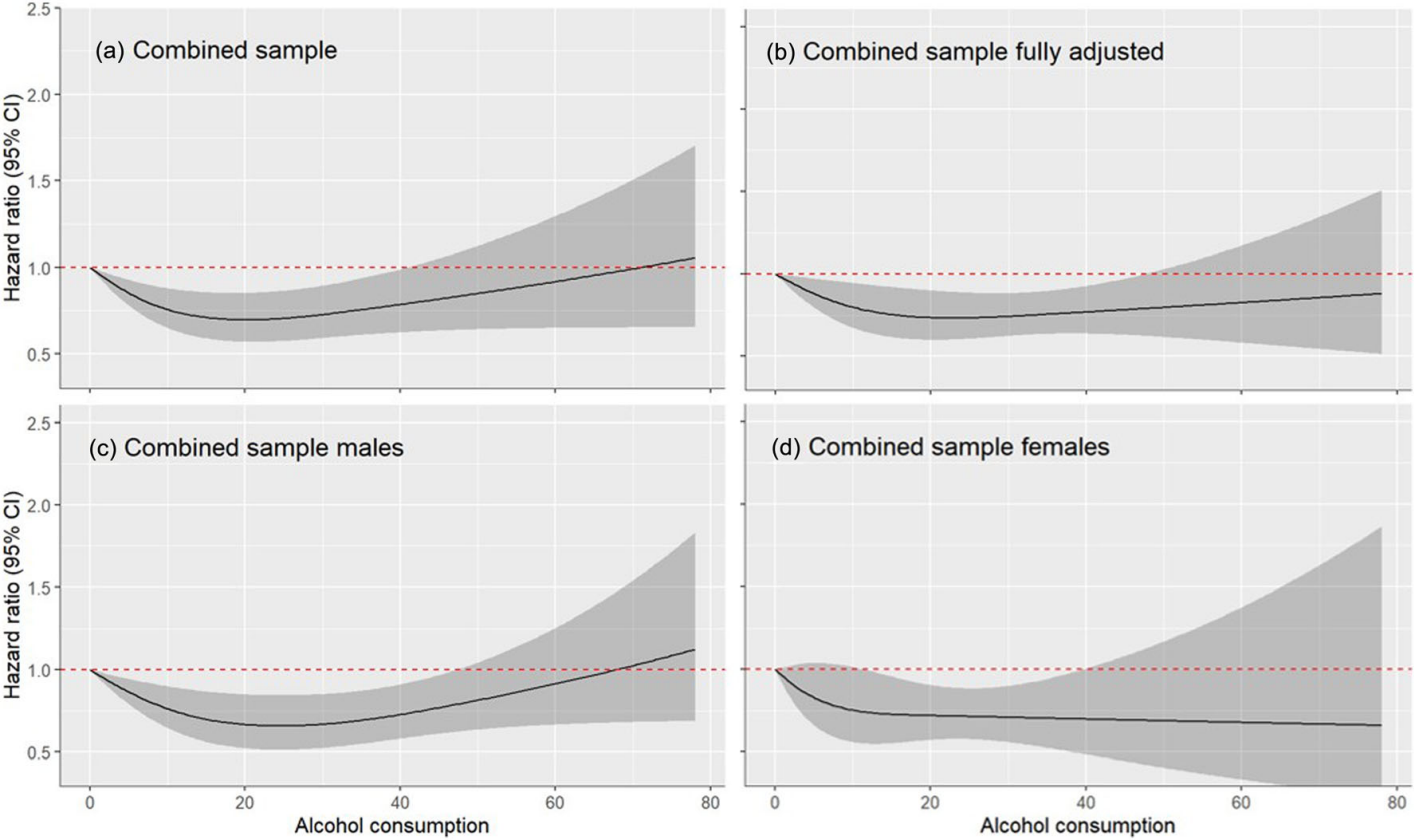
Dose–response relationship between alcohol use (g/day) and dementia including life‐time abstainers (reference group) and current drinkers

Among the 11 cohorts with data on former drinkers, 2223 participants were classified as former drinkers and excluded from the dose–response analysis with 0 g/day as the reference value (*n* = 17 964). In this analysis (Fig. 1), moderate drinking up to 40 g/day was associated with a lower risk of incident dementia when compared with life‐time abstaining (*p*‐value for non‐linearity = 0.0004). A similar relationship was identified in males (*n* = 7216; *p*‐value for non‐linearity = 0.0004) and females (*n* = 10 748; *p*‐value for non‐linearity = 0.0004), as well as in analyses which fully adjusted for demographic and clinical characteristics (*n* = 15 979; *p*‐value for non‐linearity = 0.043).

**FIGURE 2:**
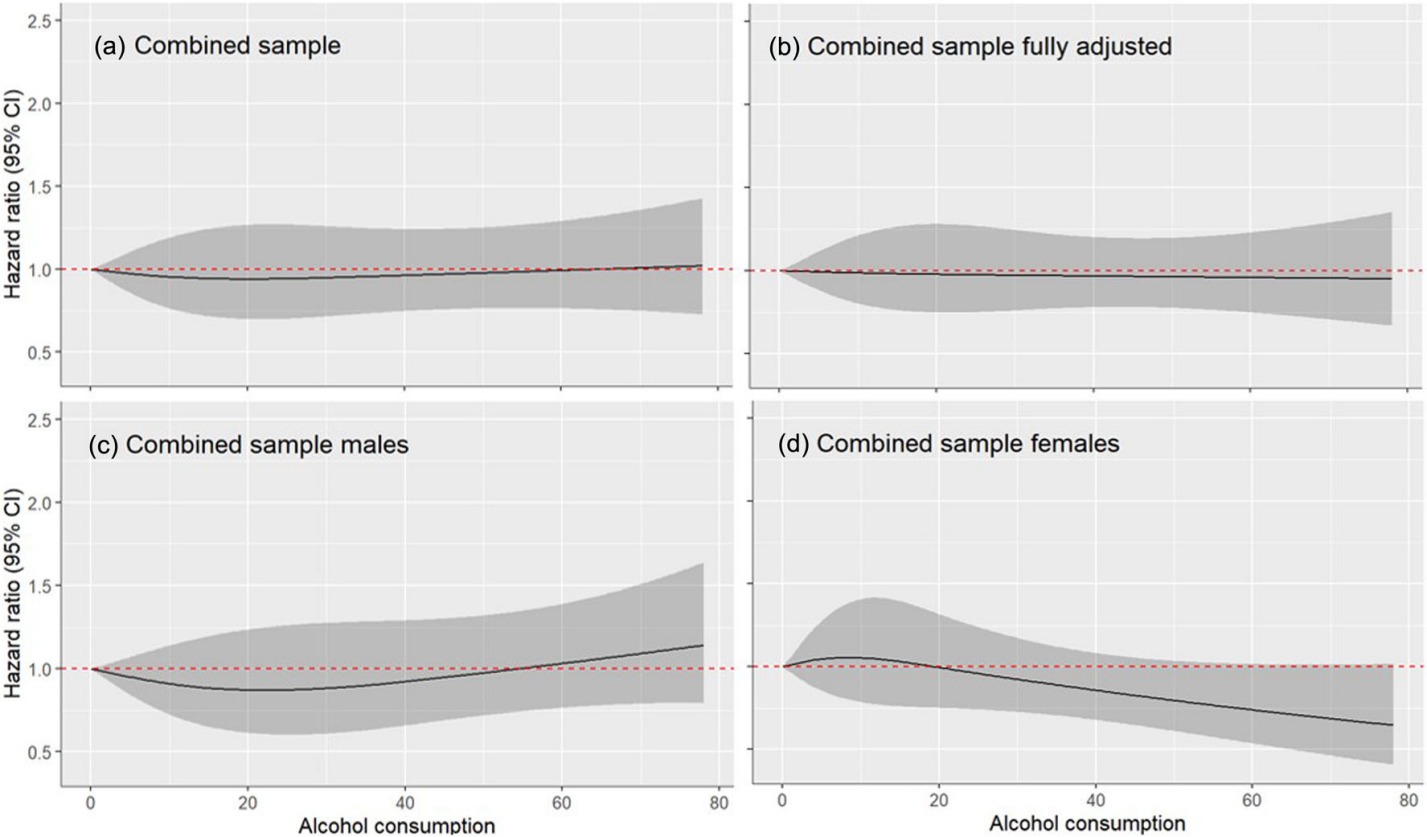
Dose–response relationship between alcohol use (g/day) and dementia among current drinkers

Meanwhile, a total of 13 335 participants (from 15 cohorts) were classified as current drinkers and included in the drinker‐only dose–response analysis. Among drinkers, there was no evidence for differences in terms of dementia risk (Fig. 2). There was also no evidence for a relationship between alcohol use and dementia risk identified in males (*n* = 7063) or females (*n* = 6272), as well as in analyses which adjusted for demographic and clinical characteristics (*n* = 11 722).

### Sensitivity analyses

Results remained similar when baseline drinking status was adjusted for using the regression dilution ratio (Supporting information, Figs S1 and S2).

In the analysis comparing daily drinkers with those who were not daily drinkers (*n* = 4581), there was no evidence for statistically significant differences in terms of dementia risk (HR = 0.64; 95% CI = 0.41–1.0; *P* = 0.052).

When those who reported stroke at baseline were excluded from the analyses, a similar relationship was identified between alcohol use and dementia risk in the samples including life‐time abstainers (*n* = 17 016) and drinkers only (*n* = 12 343; see Supporting information, Fig. S3).

Subgroup analyses by continent were possible for North America, Europe, Oceania (Australia) and Asia (Korea). For the abstainer analysis (Supporting information, Fig. S4), there were non‐linear relationships between alcohol use and dementia risk for North America (*n* = 2380), Europe (*n* = 6735) and Asia (*n* = 4737), although there was no evidence for any statistically significant differences within these reduced sample sizes. In Oceania (*n* = 2898), there was evidence of a protective effect of alcohol use across the full spectrum of consumption when compared with life‐time abstainers. For the analysis including current drinkers only, results differed across continents (Supporting information, Fig. S5). When compared to those who drank minimally (0.3 g/day), there was evidence of lower dementia risk among light–moderate drinkers in Europe (*n* = 4527) and across the full spectrum of alcohol consumption in Oceania (*n* = 2733). Conversely, in North America (*n* = 3877), there was a higher dementia risk among light–moderate drinkers when compared to those who drank minimally. Meanwhile, there was no evidence of a relationship between alcohol use and dementia risk among current drinkers in Asia (*n* = 1664).

## DISCUSSION

In a large international sample of older adults aged more than 60 years, the current study found that abstinence from alcohol is associated with an increased risk for all‐cause dementia. The increased risk associated with abstaining was evident in subsamples of both males and females, as well as in both former drinkers and life‐time abstainers. Among current drinkers in the general population, there was no consistent evidence to suggest that the amount of alcohol consumed in later life was significantly associated with dementia risk. While the current findings are relevant to the majority of older drinkers in the general population, the current study does not provide evidence on the relationships between dementia risk and heavier drinking or alcohol use disorder which are relatively rare in the general population.

### Strengths and limitations

Through the use of data harmonization and individual participant data analysis, the current study overcomes many limitations of previous research. Among the 15 cohorts, alcohol use categories were harmonized so that comparisons were consistent across cohorts. The majority of cohorts allowed the separation of current abstainers into former drinkers and life‐time abstainers, allowing the exclusion of former drinkers from the abstainer category. Within each cohort, clinical consensus diagnosis of all‐cause dementia was used as the outcome variable. Importantly, this study included cohorts from high‐income countries and low‐ and middle‐income countries (i.e. Brazil and the Republic of Congo), providing evidence of the alcohol–dementia relationship in an international context.

Balanced against these strengths, the current findings also need to be considered within the context of some limitations. Alcohol use was assessed by self‐report, which is prone to under‐reporting. Beverage type was not consistently assessed across the cohorts and therefore could not be considered in the current study. Some studies have found that some beverage types (i.e. wine) are more protective against dementia when compared with other beverage types (i.e. spirits) [37]. However, predominant beverage type is highly confounded with other socio‐demographic characteristics and some reviews have suggested that ethanol itself should be the focus of study, rather than any particular beverage type [38]. While the current study was able to account for many demographic and clinical characteristics which were harmonized across cohorts, uncontrolled confounding may still impact this study’s results. Frequency of alcohol use is likely to be an important factor in dementia risk, but the current study was limited in the way it could examine alcohol frequency across cohorts. Healthy survivor bias may also impact the current findings, particularly given the older average age of the cohorts, and possibly reflected in the small numbers of participants in the more extreme drinking categories. While data were able to be stratified to investigate the alcohol–dementia relationship in four of the six continents represented in the current study, there was insufficient power to examine this relationship in the single cohorts representing South America (Brazil) and Africa (Republic of Congo). Future work is needed to better understand the alcohol–dementia relationship in low‐ and middle‐income countries.

### Abstaining and increased dementia risk

While abstinence from alcohol has often been associated with a higher risk for dementia [37], this relationship is the subject of considerable debate. When compared with abstainers, light‐to‐moderate alcohol use has been found to be protective for vascular dementia (RR = 0.75; 95% CI: 0.57–0.98), Alzheimer's disease (RR = 0.61; 95% CI: 0.54–0.68; RR: 0.72; 95% CI: 0.61–0.86) and all‐cause dementia (RR = 0.74; 95% CI: 0.61–0.91) [10] in a previous systematic review of meta‐analyses. In a scoping review of systematic reviews, most reviews similarly found that light‐to‐moderate consumption was protective against a diagnosis of dementia, as well as death from dementia, when compared with abstinence [9]. Experimental evidence in animal models is consistent with this observational research, confirming the neurotoxicity of heavy alcohol use and the protective effects of alcohol at low doses [39, 40, 41].

It has been suggested that the increased risk of dementia associated with abstinence may be the result of including former drinkers who have ceased drinking due to other health conditions or the onset of cognitive problems [11]. Consistent with this hypothesis, previous studies have indicated that the increased risk of dementia associated with abstinence is not robust to careful control for confounding factors, particularly physical and mental health factors associated with dementia risk [42]. In the current study, however, the increased dementia risk associated with abstaining was evident after controlling for relevant demographic and clinical characteristics. In the analyses focused on categorical alcohol use, there was also no consistent difference in dementia risk for those designated as either former drinkers or life‐time abstainers. In the dose–response analysis, there was a higher dementia risk for abstainers after the exclusion of former drinkers.

While rates of abstinence varied considerably across the cohorts included in this study, the rates were high overall, and there may have been more power to identify statistically significant effects in this group when compared with current drinkers. The particularly high rates of life‐time abstaining across cohorts in the current study suggests that these data may be subject to recall and/or social desirability biases. The misidentification of former drinkers as life‐time abstainers may therefore explain some of the increased dementia risk in the abstainer group. Overall, however, there was consistent evidence from the current study to suggest that abstaining from alcohol is related to an increased dementia risk when compared to light‐moderate alcohol consumption.

Mechanisms underpinning the protective effect of light to moderate alcohol use are contested, but include indirect effects through reduced cardiometabolic disease [37] and the possible modulation of amyloid beta deposition and glymphatic function [39, 43]. While light to moderate alcohol use may reduce dementia risk, even low levels of alcohol use have been associated with reduced brain volume, grey matter atrophy and increased white matter hyperintensities [5, 44, 45], indicating that alcohol use is unlikely to be directly neuroprotective. In addition, light‐to‐moderate alcohol use has been associated with other health conditions, including some cancers [46], cautioning against recommending the commencement of alcohol use in those who abstain.

### Current drinking and dementia risk

In the combined sample, dose–response analyses focused only on current drinkers found no evidence of differences in terms of dementia risk across the spectrum of consumption that could be investigated in the current study. It should be noted that the current study does not provide evidence on heavier drinking and alcohol use disorder, which are relatively rare in population‐based observational studies of older adults. There is evidence from other sources, such as hospital‐based studies, which indicate that heavy alcohol use and alcohol use disorders are strongly and causally associated with dementia (particularly young onset dementia) [7], as well as neurocognitive diseases where alcohol use is a contributing or necessary factor (i.e. alcohol‐related dementia and Wernicke–Korsakoff syndrome) [8].

## CONCLUSION

The current study found consistent evidence to suggest that abstinence from alcohol in later life is associated with increased dementia risk internationally. Such findings need to be balanced against neuroimaging evidence suggesting that even low levels of alcohol use are associated with poorer brain health, as well as dose–response relationships between alcohol use and other health outcomes, including some cancers. For these reasons, advising those who currently abstain to initiate drinking is not recommended. Meanwhile, among current drinkers, alcohol use did not appear to be a consistent risk factor for dementia, although this relationship varied across continents and could not be examined among heavier drinkers. There is wide variability in alcohol guidelines across countries internationally, and findings from the current study support a more national‐level approach to the development of alcohol guidelines where local context can be taken into account. While other studies have demonstrated that heavy alcohol use and alcohol use disorders are strongly associated with neurocognitive disease and are key targets for preventions, the current study questions whether reducing less than heavy alcohol use in older adults aged over 60 years is an effective prevention strategy for dementia from a population‐level, or public health, perspective.

## DECLARATION OF INTERESTS

None.

## AUTHOR CONTRIBUTIONS

**Louise Rua Mewton:** Conceptualization; data curation; formal analysis; methodology; project administration. **Rachel Visontay:** Data curation; formal analysis; methodology; project administration. **Nicholas Geoffrey Hoy:** Data curation; methodology; project administration. **Darren Lipnicki:** Data curation; methodology. **Matthew Sunderland:** Methodology. **Richard Lipton:** Data curation; methodology. **Maelenn Guerchet:** Data curation; methodology. **Karen Ritchie:** Data curation; methodology. **Jenna Najar:** Data curation; methodology. **Nikolaos Scarmeas:** Data curation; methodology. **Ki Woong Kim:** Data curation; methodology. **Steffi Riedel Heller:** Data curation; methodology. **Martin van Boxtel:** Data curation; methodology. **Erin Jacobsen:** Data curation. **Henry Brodaty:** Data curation; funding acquisition; methodology; resources. **Kaarin Anstey:** Data curation; methodology. **Mary Haan:** Data curation; methodology. **Marcia Scazufca:** Data curation; methodology. **Elena Lobo:** Data curation; methodology. **Perminder Sachdev:** Data curation; methodology.

## Supporting information


**Table S1.** Details of dementia assessment in each of the included cohorts
**Table S2.** Alcohol use: study‐specific details and harmonisation protocols
**Table S3.** Smoking: Study‐specific details and harmonisation protocols
**Table S4.** Depression: Study‐specific details and harmonisation protocols
**Table S5.** Hypertension: Study‐specific details and harmonisation protocols
**Table S6.** Cholesterol: Study‐specific details and harmonisation protocols
**Table S7:** Demographic characteristics of contributing cohorts
**Table S8:** Clinical characteristics of contributing cohorts
**Table S9.** Comparison of baseline demographic and clinical characteristics of the participants included and excluded from the analyses
**Table S10.** Comparison of baseline alcohol use characteristics of participants with and without baseline dementia diagnosis
**Table S11.** Relationships between demographic and clinical covariates and dementia risk in the combined sample (n = 24 478) ^a^
**Table S12.** Combined sample hazard ratios with lifetime abstainers and former drinkers separated
**Figure S1.** Dose response relationship between usual alcohol use (grams/day; implemented using the regression dilution ratio) and dementia including lifetime abstainers (reference group) and current drinkers
**Figure S2.** Dose response relationship between usual alcohol use (grams/day; implemented using the regression dilution ratio) and dementia among current drinkers
**Figure S3.** Sensitivity analysis excluding participants reporting stroke at baseline
**Figure S4.** Sub‐group analyses by continent including lifetime abstainers (reference group) and current drinkers
**Figure S5.** Sub‐group analyses by continent including current drinkers
**Figure S1.** Love plots of covariate balance for each cohort.
