# Supplementary material for: The relationship between alcohol use and dementia in adults aged more than 60 years: a combined analysis of prospective, individual‐participant data from 15 international studies
Source: Addiction. 2022 Sep 4;118(3):412–24. doi: 10.1111/add.16035 (PMC9898084; doi:10.1111/add.16035)
Supplement: Supplementary file 1 — Table S1. Details of dementia assessment in each of the included cohorts Table S2. Alcohol use: study‐specific details and harmonisation protocols Table S3. Smoking: Study‐specific details and harmonisation protocols Table S4. Depression: Study‐specific details and harmonisation protocols Table S5. Hypertension: Study‐specific details and harmonisation protocols Table S6. Cholesterol: Study‐specific details and harmonisation protocols Table S7: Demographic characteristics of contributing cohorts Table S8: Clinical characteristics of contributing cohorts Table S9. Comparison of baseline demographic and clinical characteristics of the participants included and excluded from the analyses Table S10. Comparison of baseline alcohol use characteristics of participants with and without baseline dementia diagnosis Table S11. Relationships between demographic and clinical covariates and dementia risk in the combined sample (n = 24 478) a Table S12. Combined sample hazard ratios with lifetime abstainers and former drinkers separated Figure S1. Dose response relationship between usual alcohol use (grams/day; implemented using the regression dilution ratio) and dementia including lifetime abstainers (reference group) and current drinkers Figure S2. Dose response relationship between usual alcohol use (grams/day; implemented using the regression dilution ratio) and dementia among current drinkers Figure S3. Sensitivity analysis excluding participants reporting stroke at baseline Figure S4. Sub‐group analyses by continent including lifetime abstainers (reference group) and current drinkers Figure S5. Sub‐group analyses by continent including current drinkers Figure S1. Love plots of covariate balance for each cohort. [file ADD-118-412-s001.docx]

Table S1. Details of dementia assessment in each of the included cohorts

| **Cohort** | **Dementia assessment details** |
| --- | --- |
| EAS | Cognitive function, including performance on various neuropsychological tests measuring different cognitive domains was completed. Subjective memory impairment and functional decline were also assessed. If possible, informant reports of cognitive impairment and functional decline were obtained. Clinical diagnoses were determined according to DSM-IV criteria and assigned at consensus case conferences which included the study neurologist and the study neuropsychologist who comprehensively reviewed cognitive test results, relevant neurological signs and symptoms, and assessments of functional status. A similar procedure was adopted for all follow-up assessments. To ensure that diagnostic criteria were uniform over time, all individuals evaluated before the release of DSM-IV in 1994 were retrospectively reconferenced according to DSM-IV criteria. |
| EPIDEMCA | Cognitive disorders were assessed at baseline and follow-up assessments through a two-phase screening and diagnostic procedure. The Community Screening Interview for Dementia (CSI-D) was administered to identify suspected cases of dementia. All participants obtaining a poor performance on the CSI-D (COGSCORE ≤ 24.5) were referred for detailed clinical assessment with a neurologist. A neurological examination was performed. Orientation skills and daily living activities were also investigated to evaluate dependence. Neurological assessments also involved additional cognitive tests. Consensus diagnosis was determined according to DSM-IV criteria and based on reviewing of all medical records and performance on clinical assessments and cognitive tests. |
| ESPRIT | A neurologist examined all participants at baseline using a standardised clinical interview, as well as a battery of cognitive tests, to identify both dementia and mild cognitive impairment. A panel of expert neurologists further validated all incident cases of dementia independently from the study investigators. Diagnoses were determined according to DSM-IV criteria. The same procedure was followed for all follow-up assessments. |
| Framingham | In the 1975 assessment, individuals with low cognitive scores according to neuropsychological testing (lowest 10%) underwent further neurologic assessment and then a dementia-free cohort was established that only included individuals without dementia. From 1981, individuals who scored below prespecified cut-offs on the MMSE (adjusted for educational level and prior performance) were subjected to further evaluation at each assessment. Detailed evaluation consisted of an examination by a neurologist and a neuropsychologist. Identified dementia cases were sent to a review committee, which made the final decision regarding the presence of dementia in accordance with DSM-IV criteria. This review committee comprised at least two neurologists and one or more neuropsychologists and used data obtained during neurologic and neuropsychological evaluation, information from primary care physicians, hospitalization records, brain imaging and data obtained by telephone interview of next of kin to arrive at a clinical consensus. |
| H70 | Dementia was diagnosed according to DSM-III-R criteria, using information from neuropsychiatric examination, including a battery of cognitive tests, as well as a semi-structured close informant interview. The neuropsychiatric examinations and the close informant interviews were performed by medical doctors, psychologists, or psychiatric research nurses. At each assessment occasion, dementia status was determined based on computerized algorithms and a consensus conference including at least two psychiatrists. |
| HELIAD | Certified neurologists and trained neuropsychologists administered structured questionnaires and conducted neuropsychological assessments. Trained psychometricians administered a battery of neuropsychological tests to assess major cognitive domains. Information collected from all evaluations was reviewed at expert consensus meetings, including the neurologists who examined the participants clinically and neuropsychologists. Dementia diagnoses were made in accordance with DSM-IV- criteria, using the same procedure at each assessment |
| KLOSCAD | At each assessment occasion, geriatric neuropsychiatrists with expertise in dementia research administered a standardized diagnostic interview, as well as physical and neurological examinations. Subjective memory complaints were assessed, and informant interviews were conducted to assess subjective complaints associated with cognitive impairment. A battery of cognitive tests was administered. Behavioural and psychological symptoms of dementia, activities of daily living and level of dependency were also evaluated. A panel of research neuropsychiatrists determined the final diagnoses in accordance with DSM-IV criteria. |
| LEILA | At each assessment occasion, a fully structured interview was administered to the study subjects by trained physicians and psychologists. A battery of cognitive tests was administered. Additional information on cognitive and psychosocial functioning was obtained through fully structured informant interviews. Consensus conferences were held to determine dementia status in accordance with DSM-IV criteria. |
| MAAS | At baseline, participants were excluded based on an existing diagnosis of dementia or a score below 24 on the Mini-Mental State Examination (MMSE),. Included participants were assessed on a battery of cognitive tests, as well as activities of daily living and subjective cognitive complaints. A general practitioner and the investigators identified cases of dementia in accordance with DSM-III-R/DSM-IV criteria using all available clinical information and neuropsychological test data at all follow up occasions |
| MYHAT | At baseline, trained interviewers administered the MMSE (corrected for age and education). Individuals scoring less than 21 of 30 were classified as moderately or severely impaired and not assessed further. Included participants received detailed clinical and neuropsychological assessments at baseline and all follow-up assessments. Dementia was assessed through a purely cognitive classification and through assessment of everyday functioning (using the CDR). For cognitive classification, participants received a battery of tests assessing numerous cognitive domains. Composite scores for each cognitive domain were created. Normative reference points were created for each domain (excluding participants with CDR>1). On the basis of these reference points, participants were classified as dementia cases if composite scores in at least two domains were >2.0 SD below the mean for the individual's reference group. The CDR was administered by trained and certified interviewers and used to classify dementia cases based solely on measures of everyday functioning. CDR >1 was used to determine the presence of dementia and classification for each participant was finalized by consensus among 2 or more interviewers, ignoring the neuropsychological data but determining that the reported or observed functional impairments were attributable to cognitive difficulties. |
| PATH | For each assessment, clinical diagnosis of dementia was conducted in two phases. In Phase 1, all participants were screened for cognitive disorders and were selected for further assessment if they scored ≤25 on the MMSE or below the 5^th^ percentile on one of numerous cognitive tests. In the second phase, those who screened positive in Phase 1 were given a Structured Clinical Assessment for Dementia by one of two physicians, a neuropsychological assessment (incorporating numerous additional cognitive tests), and the CDR Scale. An informant was also interviewed where possible. Diagnoses were made by consensus clinical judgment in accordance with DSM-IV criteria. |
| SALSA | Cognitive screening tests were used to determine the need for further neuropsychological evaluation. Participants received formal neuropsychological evaluation if adjusted screening scores fell below the 20th percentile on any adjusted test. Formal neuropsychological evaluation included administration of a battery of cognitive tests, as well as the Informant Questionnaire on Cognitive Decline in the Elderly. Participants were referred for a neurological examination if they met at least one of the following criteria: (1) a score below the 10th per-centile on one or more of the six neuropsychological tests and a score on the IQCODE of 3.40 or greater (2) impaired scores on four or more neuropsychological tests regardless of IQCODE, or (3) IQCODE greater than 4.0. A team of neurologists and a neuropsychologist determined all dementia cases via consensus and in accordance with DSM-IV criteria at each assessment occasion |
| SPAH | The assessment of dementia was carried out with a harmonized one-phase dementia diagnostic procedure developed by the 10/66 Dementia Research Group and a detailed assessment of the onset and course of the dementia syndrome. This procedure included the Community Screening Instrument for Dementia (CSI-D), a battery of cognitive tests and a structured neurological assessment. An interview with the informants assessed participant’s cognitive and daily function and a brief history of the participant’s functional and cognitive decline. Diagnosis of dementia was made in accordance with DSM-IV criteria and based on all information collected from the subject and his/her informant. A similar procedure was followed for each assessment occasion. |
| Sydney MAS | At baseline, participants were excluded if they had a previous diagnosis of dementia, if they had a MMSE score of < 24 (adjusted for age, education, and non-English speaking background at study entry), or if they received a diagnosis of dementia after comprehensive baseline assessments. A comprehensive neuropsychological battery was administered to assess numerous cognitive domains. Individuals who had: a score of at least 1.5 standard deviations below published normative data on a memory and a non-memory measure, or on two non-memory measures, or reduced neuropsychological scores and a decline in activities of daily living based on an informant interview were further assessed. Consensus diagnoses were made in accordance with DSM-IV criteria, by an expert team comprising neuropsychiatrists, psycho-geriatricians, and neuropsychologists, on the basis of the available clinical, neuropsychological, laboratorial and imaging data. A similar procedure was followed for each assessment occasion. |
| ZARADEMP | A two-phase epidemiological case-finding process for dementia was implemented in the baseline study and a similar method in the follow up waves. In phase I, well-trained and regularly supervised lay interviewers conducted the ZARADEMP interview, including numerous cognitive tests and screening instruments. Medical reports and laboratory data were consulted to complete the data. Outside caregivers were interviewed when the participant was considered unreliable. Participants were considered probable cases based on standard cut-off points used for the Geriatric Mental State and/or the MMSE. In phase II, the probable cases of dementia were reassessed by research psychiatrists. The same assessment instruments and methods were used and a neurological examination was performed. Identified cases of dementia were presented to a panel of four research psychiatrists for final diagnostic decisions, which were made in accordance with DSM-IV criteria. |

*Note.* EAS, Einstein Aging Study; EPIDEMCA, Epidemiology of Dementia in Central Africa; ESPRIT, Etude Santé Psychologique Prévalence Risques et Traitement; FHS, Framingham Heart Study; H70, Gothenburg H70 Birth Cohort Studies; HELIAD, Hellenic Longitudinal Investigation of Aging and Diet; KLOSCAD, Korean Longitudinal Study on Cognitive Aging and Dementia; LEILA75+, Leipzig Longitudinal Study of the Aged; MAAS, Maastricht Ageing Study; MYHAT, Monongahela-Youghiogheny Healthy Aging Team; PATH, Personality and Total Health Through Life Project; SALSA, Sacramento Area Latino Study on Aging; SPAH, São Paulo Ageing and Health Study; Sydney MAS, Sydney Memory and Ageing Study; ZARADEMP, Zaragoza Dementia Depression Project.

**Alcohol use**

Using the variable information provided within each cohort (Table S2), units of alcohol were assumed to be standard drinks and converted into number of grams of alcohol per day (based on the number of grams in a standard drink within each country). This grams per day variable was then categorised into both the three-level and five-level alcohol use variables described in text.

Table S2. Alcohol use: study-specific details and harmonisation protocols

| **Cohort** | **Indicators of alcohol use at baseline** |
| --- | --- |
| EAS | Have you had at least one drink of beer, wine or liquor during the past year? (0=No; 1=Yes).  Quantity to use/period to use (continuous/month).  During the year, on average, how often did you drink alcoholic beverages, beer/wine/liquor? (continuous, participants nominate whether per day,  week, month or year).  During the year, on average, how many cans or bottles of beer did you drink? (continuous, participants nominate whether they report per day, week,  month or year).  During the year, on average, how many glasses of wine did you drink? (continuous, participants nominate whether they report per day, week,  month or year).  During the year, on average, how many glasses of wine did you drink? (continuous, participants nominate whether they report per day, week,  month or year).  During the year, on average, how many shots or drinks or hard liquor did you drink? (continuous, participants nominate whether they report per day,  week, month or year).  What was the usual number of drinks of alcohol you had per week when you were less than 20 years old? (1=None; 2=Less than 3 per week; 3= 3 to 6 per week; 4=7 to 13 per week; 5=More than 14 per week).  What was the usual number of drinks of alcohol you had per week when you were between 20 and 50 years old? (1=None; 2=Less than 3 per week;  3= 3 to 6 per week; 4=7 to 13 per week; 5=More than 14 per week).  What was the usual number of drinks that you had per week when you were over 50 years old (1=None; 2=Less than 3 per week; 3= 3 to 6 per  week; 4=7 to 13 per week; 5=More than 14 per week). |
| EPIDEMCA | Alcohol consumption (0=none; 1=sometimes; 2= regularly [at least 5 days/week]; 9= don’t know).  Number of units of beer/week (continuous).  Number of units of liquor/week (continuous).  Number of units of ‘local beverages (i.e., palm wine)/week (continuous). |
| ESPRIT | Alcohol consumption (1=current; 2=never; 3=past).  Number of alcoholic drinks per week (continuous). |
| Framingham | Beer – bottles/cans/glasses per week (continuous).  Wine – glasses per week (continuous).  Cocktails/straight drinks per week (continuous). |
| H70 | Historical alcohol use (0=alcohol consumer; 1 = alcohol abstainer for 0-1 years; 2= alcohol abstainer 1-2 years; 3 = alcohol abstainer for 2-5 years; 4 = alcohol abstainer for 6-10 years; 5 = alcohol abstainer for 11-15 years; 6 = alcohol abstainer for more than 15 years; 7 = lifetime alcohol abstainer).  Frequency of drinking beer, past month (0 = do not use alcohol; 1 = no beer; 2 = 1-2 times per week; 3 = 3-5 times per week; 4 = mostly every day light beer, less than one bottle; 5 = mostly every day, stronger beer, less than one bottle; 6 = mostly daily light beer, one bottle or more; 7 = mostly daily stronger beer, one bottle or more).  Frequency of drinking wine, past month (0 = do not use alcohol; 1 = no wine; 2 = less than once per week; 3 = 1-2 times per week; 4 = 3-5 times  per week; 5 = almost daily or daily).  Frequency of drinking liquor, past month (0 = do not use alcohol; 1 = no liquor; 2 = less than once a week; 3 = 1-2 times per week; 4 = 3-5 times  per week; 5 = almost daily or daily).  Estimated total consumption/grams per week (0=0; 1=0-20; 2=20-40; 3=40-60; 4=60-100; 5=100-150; 6=150-250; 7=250-500; 8=More than 500). |
| HELIAD | Current drinking of alcohol (0=No; 1=Yes).  Past drinking of alcohol (0=No; 1=Yes).  Number of glasses -Tsipuro (continuous).  Frequency of Tsipouro (0=twice or more per day; 1 = 5, 6, or 7 times a week; 2 = 3-4 times a week; 3 = 1-5 times a month; 4 = less than once a month; 5 = once a year or rarer; 99=not applicable).  Number of glasses – beer (continuous).  Frequency of beer (0=twice or more per day; 1 = 5, 6, or 7 times a week; 2 = 3-4 times a week; 3 = 1-5 times a month; 4 = less than once a month; 5 = once a year or rarer; 99=not applicable).  Number of glasses – wine (continuous).  Frequency of wine (0=twice or more per day; 1 = 5, 6, or 7 times a week; 2 = 3-4 times a week; 3 = 1-5 times a month; 4 = less than once a month; 5 = once a year or rarer; 99=not applicable).  Number of glasses – whisky (continuous).  Frequency of whisky (0=twice or more per day; 1 = 5, 6, or 7 times a week; 2 = 3-4 times a week; 3 = 1-5 times a month; 4 = less than once a month; 5 = once a year or rarer; 99=not applicable). |
| KLOSCAD | Amount of alcoholic drinks consumed in standard units per week (continuous).  Lifetime alcohol consumption in standard units per week (continuous).  Duration of lifetime alcohol consumption in years (continuous).  Total amount of lifetime alcohol consumption in standard units (continuous).  Age at beginning of alcohol consumption in years (continuous).  Age began abstaining from alcohol in years (continuous). |
| LEILA | Ever drank alcohol? (0=No; 1=Yes).  How often do you drink alcohol (used for frequency analysis; 0=less than once a month; 1=once a month; 2=twice a month; 3=three times a month; 4=once a week; 5=twice a week; 6=three times a week; 7=four times a week; 8=five times a week; 9=six times a week; 10=daily).  Drinks per day? (continuous). |
| MAAS | Drink alcohol? (0=No; 1=Yes).  Drinks per day? (used for frequency analysis; 0=1-2 units; 1=3-6 units; 2=7-10 units; 3=more than 10 units; 4=N/A).  Days drinking per week? (0= less than 1; 1=1-2 days; 2=3-4 days; 3=5-6 days; 4=every day; 5=N/A). |
| MYHAT | Ever drink? (0=Never; 1=More than 1 year ago; 2=Within past year).  Frequency (used for frequency aanlysis; 0=N/A; 1=Daily; 2=[not daily] more than weekly; 3=once a week; 4=not weekly but more than once a month; 5 =once a month (or less).  Amount (0=Does not currently [past year]; 1=1 drink; 2=2-3 drinks; 3=4-5 drinks; 4 = 6 or more drinks). |
| PATH | Alcohol frequency (1=Never; 2=Not in the last year; 3=Monthly or less; 4=2 to 4 times a month; 5=2 or 3 times a week; 6= 4 or more times a week).  Number of drinks per day (1=1 or 2; 2 =3 or 4; 3= 5 or 6; 4=7 or 9; 5=10 or more).  Ever drunk alcohol (1=Yes; 2=No). |
| SALSA | Drink beer? (1=Yes; 2=No; 98=Don’t know; 99=Refused).  How often drink beer? (1=Daily; 2=Weekly; 3=Monthly; 4=Yearly; 5=Rarely/Never; 98=Don’t know; 99=Refused).  How much beer? (continuous).  Drink wine? (1=Yes; 2=No; 98=Don’t know; 99=Refused).  How often drink wine? (1=Daily; 2=Weekly; 3=Monthly; 4=Yearly; 5=Rarely/Never; 98=Don’t know; 99=Refused).  How much wine? (continuous).  Drink hard liquor? (1=Yes; 2=No; 98=Don’t know; 99=Refused).  How often drink hard liquor? (1=Daily; 2=Weekly; 3=Monthly; 4=Yearly; 5=Rarely/Never; 98=Don’t know; 99=Refused).  How much hard liquor? (continuous).  Number of alcoholic drinks per week (continuous). |
| SPAH | Units of alcohol drunk per week before age of 65 (continuous).  Units of alcohol drunk per week now (continuous). |
| Sydney MAS | How often have you drink alcohol in the last year (used for frequency analysis; 1= Not in the last year; 2 = monthly or less; 3 = 2-4 times a month; 4 = 2-3 times a week; 5 = 4-6 times a week; 6 = daily; 777=N/A).  Have you ever drunk alcohol (1=No; 2=Yes; 777=N/A; 888=Don’t know).  How many standard drinks do you have on a typical day (1=1; 2=2 or 3; 3=4 or 5; 4=6 or 7; 5=8 or more; 777=N/A). |
| ZARADEMP | Alcohol consumption status (0=Never; 1=Habitual; 2=Occasional; 3=Ex-drinker).  Daily intake of wine in the last year (continuous).  Weekend intake of wine in the last year (continuous).  Daily intake of beers in the last year (continuous).  Weekend intake of beers in the last year (continuous).  Daily intake of spirits in the last year (continuous).  Weekend intake of spirits in the last year (continuous). |

*Note.* EAS, Einstein Aging Study; EPIDEMCA, Epidemiology of Dementia in Central Africa; ESPRIT, Etude Santé Psychologique Prévalence Risques et Traitement; FHS, Framingham Heart Study; H70, Gothenburg H70 Birth Cohort Studies; HELIAD, Hellenic Longitudinal Investigation of Aging and Diet; KLOSCAD, Korean Longitudinal Study on Cognitive Aging and Dementia; LEILA75+, Leipzig Longitudinal Study of the Aged; MAAS, Maastricht Ageing Study; MYHAT, Monongahela-Youghiogheny Healthy Aging Team; PATH, Personality and Total Health Through Life Project; SALSA, Sacramento Area Latino Study on Aging; SPAH, São Paulo Ageing and Health Study; Sydney MAS, Sydney Memory and Ageing Study; ZARADEMP, Zaragoza Dementia Depression Project.

Table S3. Smoking: Study-specific details and harmonisation protocols

| **Cohort** | **Indicators of smoking at baseline** |
| --- | --- |
| EAS | If ‘no’ to ‘smoked at least 100 cigarettes in your lifetime’, Smoking status = 1; if ‘yes’ to ‘smoked at least 100 cigarettes  in lifetime’ and ‘no’ to ‘current smoker’, Smoking status = 2; if ‘yes’ to ‘current smoker’ and ‘yes’ to  ‘smoked at least 100 cigarettes in your lifetime’, Smoking status = 3; if ‘yes’ to ‘smoked at least 100 cigarettes in your  lifetime’ and missing data for current smoker, Smoking status = missing; if missing data for ‘smoked at least 100 cigarettes  in your lifetime, Smoking status = missing.^1^ |
| EPIDEMCA | Smoking variable already categorised as needed (i.e. never, former and current). |
| ESPRIT | Smoking variable already categorised as needed (i.e. never, former and current). |
| Framingham | If ‘non-smoker’ to ‘ever smoked cigarettes’, Smoking status = 1; if ‘yes, formerly’ to ‘ever smoked’, smoking status = 2;  if ‘yes, currently’ to ‘ever smoked’ or ‘yes’ to ‘do you now smoke pipes’ or ‘yes’ to ‘do you now smoke cigars’,  Smoking status = 3. |
| H70 | Smoking variable already categorised as needed (i.e. never, former and current). |
| HELIAD | If ‘no’ to ‘current smoking’ and ‘no’ to ‘smoking in the past’, Smoking status = 1; if ‘no’ to ‘current smoking’ and ‘yes’ to  ‘smoking in the past’, Smoking status = 2; if ‘yes’ to ‘current smoking’, Smoking status = 3. |
| KLOSCAD | If current amount of smoking packs per day (continuous) > 0, Smoking status =3; if lifetime amount of smoking packs  day (continuous) = 0, Smoking status = 1; If age at the cessation of smoking (continuous) > 0, Smoking status = 2. |
| LEILA | If ‘no’ to ‘ever smoked’, Smoking status = 1; if ‘yes’ to ‘smoking was quitted’, Smoking status =2; if ‘no’ to ‘smoking  was quitted’, Smoking status =3. |
| MAAS | Smoking variable already categorised as needed (i.e. never, former and current). |
| MYHAT | If ‘no’ to ‘ever smoke’, Smoking status =1; if ‘yes’ to ‘ever smoke’ and ‘no’ to ‘smoke now’, Smoking status = 2; if ‘yes’  To ‘smoke now’, Smoking status = 3. |
| PATH | If ‘no’ to ‘smoke past’, Smoking status = 1; if ‘no’ to ‘smoke now’ and ‘yes’ to ‘smoke past’, Smoking status = 2; if ‘yes’  to ‘smoke now’, Smoking status =3. |
| SALSA | Smoking variable already categorised as needed. |
| SPAH | If ‘no’ to ‘ever smoked’, Smoking status = 1; if ‘yes’ to ‘ever smoked’ and ‘no’ to ‘still smoking’, Smoking status = 2; if  ‘yes’ to ‘still smoking’, Smoking status = 3. |
| Sydney MAS | If ‘no’ to ‘ever smoke tobacco’, Smoking status = 1; if ‘yes’ to ‘ever smoke tobacco’ and ‘age stopped smoking’  (continuous) > 0, Smoking status = 2; if ‘yes’ to ‘ever smoke tobacco’ and ‘not applicable’ to ‘age stopped smoking’,  Smoking status = 3. |
| ZARADEMP | Smoking variable already categorised as needed (i.e. never, former and current). |

*Note.* EAS, Einstein Aging Study; EPIDEMCA, Epidemiology of Dementia in Central Africa; ESPRIT, Etude Santé Psychologique Prévalence Risques et Traitement; FHS, Framingham Heart Study; H70, Gothenburg H70 Birth Cohort Studies; HELIAD, Hellenic Longitudinal Investigation of Aging and Diet; KLOSCAD, Korean Longitudinal Study on Cognitive Aging and Dementia; LEILA75+, Leipzig Longitudinal Study of the Aged; MAAS, Maastricht Ageing Study; MYHAT, Monongahela-Youghiogheny Healthy Aging Team; PATH, Personality and Total Health Through Life Project; SALSA, Sacramento Area Latino Study on Aging; SPAH, São Paulo Ageing and Health Study; Sydney MAS, Sydney Memory and Ageing Study; ZARADEMP, Zaragoza Dementia Depression Project.

Table S4. Depression: Study-specific details and harmonisation protocols

| **Cohort** | **Indicators of depression at baseline** |
| --- | --- |
| EAS | Geriatric Depression Scale (GDS; 15-item); a score of 6+ was coded as depression present |
| EPIDEMCA | Geriatric Mental State-Automated Geriatric Examination for Computer Assisted Taxonomy (GMS-AGECAT);  a score of 3+ was coded as depression present |
| ESPRIT | Centre for Epidemiological Studies Depression Scale (CES-D); a score of 16+ was coded as depression present |
| Framingham | Currently taking anti-depressants |
| H70 | Diagnostic and Statistical Manual of Mental Disorders Fifth Edition (DSM-5) rating based on the Montgomery  -Åsberg Depression Rating Scale (MADRS) |
| HELIAD | Geriatric Depression Scale (GDS; 15-item); a score of 6+ was coded a depression present |
| KLOSCAD | Korean Geriatric Depression Scale (GDS-KR; 30-item); a score of 16+ was coded as depression present |
| LEILA | Centre for Epidemiological Studies Depression Scale (CES-D); a score of 16+ was coded as depression present |
| MAAS | Upper quartile of the 90-item Symptom Checklist (SCL-90) for males and females separately |
| MYHAT | Modified Centre for Epidemiological Studies Depression Scale (CES-D); 90^th^ percentile; a score of 3+ was coded as  depression present |
| PATH | Goldberg Anxiety and Depression Scale - Depression; a score of 6+ was coded as depression present |
| SALSA | Centre for Epidemiological Studies Depression Scale (CES-D); a score of 16+ was coded as depression present |
| SPAH | International Classification of Diseases (10th revision; ICD-10) rating based on Geriatric Mental State (GMS) and Neuropsychiatric Inventory (NPI) items covering the past month: mild (1), moderate (2), or severe (3) major depression (vs 0) |
| Sydney MAS | Geriatric Depression Scale (GDS; 15-item); a score of 6+ was coded as depression present |
| ZARADEMP | Geriatric Mental State-Automated Geriatric Examination for Computer Assisted Taxonomy (GMS-AGECAT); a score  of 3+ was coded as depression present |

*Note.* EAS, Einstein Aging Study; EPIDEMCA, Epidemiology of Dementia in Central Africa; ESPRIT, Etude Santé Psychologique Prévalence Risques et Traitement; FHS, Framingham Heart Study; H70, Gothenburg H70 Birth Cohort Studies; HELIAD, Hellenic Longitudinal Investigation of Aging and Diet; KLOSCAD, Korean Longitudinal Study on Cognitive Aging and Dementia; LEILA75+, Leipzig Longitudinal Study of the Aged; MAAS, Maastricht Ageing Study; MYHAT, Monongahela-Youghiogheny Healthy Aging Team; PATH, Personality and Total Health Through Life Project; SALSA, Sacramento Area Latino Study on Aging; SPAH, São Paulo Ageing and Health Study; Sydney MAS, Sydney Memory and Ageing Study; V1, Variable 1; V2, Variable 2; V3, Variable 3; ZARADEMP, Zaragoza Dementia Depression Project.

Table S5. Hypertension: Study-specific details and harmonisation protocols

| **Cohort** | **Indicators of hypertension at baseline ^a^** |
| --- | --- |
| EAS | Blood pressure and/or medication |
| EPIDEMCA | Blood pressure |
| ESPRIT | Blood pressure |
| Framingham | Blood pressure and/or medication |
| H70 | High blood pressure and/or medication |
| HELIAD | Self-report |
| KLOSCAD | Self-report |
| LEILA | Blood pressure and/or self-report |
| MAAS | Blood pressure |
| MYHAT | Blood pressure and/or medication and/or self-report |
| PATH | Blood pressure and/or medication and/or self-report |
| SALSA | Blood pressure and/or medication and/or self-report |
| SPAH | Blood pressure and/or medication |
| Sydney MAS | Blood pressure and/or self-report |
| ZARADEMP | Self-report |

^a^ High blood pressure criteria are seated systolic blood pressure ≥140 mmHg or diastolic blood pressure ≥90 mmHg

*Note.* EAS, Einstein Aging Study; EPIDEMCA, Epidemiology of Dementia in Central Africa; ESPRIT, Etude Santé Psychologique Prévalence Risques et Traitement; FHS, Framingham Heart Study; H70, Gothenburg H70 Birth Cohort Studies; HELIAD, Hellenic Longitudinal Investigation of Aging and Diet; KLOSCAD, Korean Longitudinal Study on Cognitive Aging and Dementia; LEILA75+, Leipzig Longitudinal Study of the Aged; MAAS, Maastricht Ageing Study; MYHAT, Monongahela-Youghiogheny Healthy Aging Team; PATH, Personality and Total Health Through Life Project; SALSA, Sacramento Area Latino Study on Aging; SPAH, São Paulo Ageing and Health Study; Sydney MAS, Sydney Memory and Ageing Study; V1, Variable 1; V2, Variable 2; V3, Variable 3; ZARADEMP, Zaragoza Dementia Depression Project.

Table S6. Cholesterol: Study-specific details and harmonisation protocols

| **Cohort** | **Indicators of cholesterol at baseline ^a^** |
| --- | --- |
| EAS | Cholesterol |
| EPIDEMCA | Cholesterol |
| ESPRIT | Cholesterol and/or triglycerides and/or medication |
| Framingham | Cholesterol and/or triglycerides |
| H70 | Cholesterol |
| HELIAD | Self-report |
| KLOSCAD | Self-report |
| LEILA | - |
| MAAS | Medication |
| MYHAT | Cholesterol and/or triglycerides and/or medication |
| PATH | Medication |
| SALSA | Cholesterol and/or triglycerides and/or medication |
| SPAH | Cholesterol and/or triglycerides |
| Sydney MAS | Cholesterol and/or triglycerides and/or medication and/or self-report |
| ZARADEMP | Self-report |

^a^ Criteria for cholesterol are ≥240mg/dL or >6.2mmol/L, and triglycerides ≥200mg/dL or >2.3mmol/L.

*Note.* EAS, Einstein Aging Study; EPIDEMCA, Epidemiology of Dementia in Central Africa; ESPRIT, Etude Santé Psychologique Prévalence Risques et Traitement; FHS, Framingham Heart Study; H70, Gothenburg H70 Birth Cohort Studies; HELIAD, Hellenic Longitudinal Investigation of Aging and Diet; KLOSCAD, Korean Longitudinal Study on Cognitive Aging and Dementia; LEILA75+, Leipzig Longitudinal Study of the Aged; MAAS, Maastricht Ageing Study; MYHAT, Monongahela-Youghiogheny Healthy Aging Team; PATH, Personality and Total Health Through Life Project; SALSA, Sacramento Area Latino Study on Aging; SPAH, São Paulo Ageing and Health Study; Sydney MAS, Sydney Memory and Ageing Study; V1, Variable 1; V2, Variable 2; V3, Variable 3; ZARADEMP, Zaragoza Dementia Depression Project.

**Additional information on covariate harmonisation**

**Education**

Sufficient data for education was available for 14 of the included studies, measured as a continuous variable (i.e., years of education) in 13 studies and as a categorical variable in one cohort (i.e. Framingham). For Framingham, education categories included: none; fourth grade or less; fifth, sixth, or seventh grade; grade school graduate; high school, not graduate; high school graduate; college, not graduate; college, graduate; postgraduate; and business college/nursing school/music school/art school. The education variable was converted to continuous by recoding categorical variables as follows: none = 0 years education; fourth grade or less = 2 years education; fifth, sixth, or seventh grade = 6 years education; grade school graduate = 8 years education; high school, not graduate = 10 years education; high school graduate = 12 years education; college, not graduate = 14 years education; college, graduate = 16 years education; postgraduate = 18 years; and business college/nursing school/music school/art school = 12 years.

**Body mass index**

For EAS, BMI data was collected through standard assessment and separately through a phone exam. The standard assessment of BMI was used for the present study but if participants were missing data for that variable, data from the phone exam was used as an alternative. No BMI data was available for PATH; however, participant BMI was able to be calculated using data available for weight and height (i.e., BMI=kg/m^2^).

**History of stroke, diabetes and myocardial infarction**

For most cohorts, history of stroke, diabetes and myocardial infarction was based on self-report (yes/no) of the event. For HELIAD, the stroke variable represents self-reported history of stroke and/or transient ischemic attack. For PATH, participants were not asked about myocardial infarction specifically, but were asked whether they had experienced ‘heart trouble’.

Table S7: Demographic characteristics of contributing cohorts

|  | Education (Years) | | BMI | | Smoking status | | | |
| --- | --- | --- | --- | --- | --- | --- | --- | --- |
| Cohort (n) | Mean (SD) | Missing % (n) | Mean (SD) | Missing % (n) | Current % (n) | Former % (n) | Never % (n) | Missing % (n) |
| EAS (1284) | 13.7 (3.6) | - | 28.1 (5.3) | 52.5 (674) | 6.8 (87) | 46.8 (601) | 45.2 (581) | 1.2 (15) |
| EPIDEMCA (721) | - | 100 (721) | 21.7 (4.9) | 4.4 (32) | 13.3 (96) | 5.8 (42) | 80.6 (581) | 0.2 (2) |
| ESPRIT (1917) | 10.4 (3.8) | 0.1 (1) | 25.0 (3.5) | 0.6 (13) | 6.4 (123) | 35.6 (681) | 58.0 (1112) | 0.1 (1) |
| FHS (1658) | 11.5 (3.0) | 3.0 (49) | 26.6 (4.3) | 3.1 (51) | 16.3 (271) | 31.8 (527) | 50.1 (831) | 1.7 (29) |
| H70 (576) | 9.9 (4.3) | 3.1 (18) | 26.7 (4.1) | 2.8 (16) | 10.4 (60) | 28.8 (166) | 40.8 (235) | 20.0 (115) |
| HELIAD (954) | 7.8 (4.8) | 2.4 (23) | 29.1 (4.4) | 1.5 (14) | 10.2 (97) | 26.8 (256) | 62.8 (599) | 0.2 (2) |
| KLOSCAD (5139) | 8.3 (5.3) | 0.02 (1) | 24.1 (3.0) | 7.6 (387) | 8.2 (422) | 21.7 (1113) | 69.8 (3589) | 0.3 (15) |
| LEILA75+ (851) | 11.9 (1.8) | 1.9 (16) | - | 100 (851) | 1.8 (15) | 24.7 (210) | 68.3 (581) | 5.3 (45) |
| MAAS (433) | 9.5 (2.8) | - | 27.7 (3.8) | - | 19.6 (85) | 43.6 (189) | 36.7 (159) | - |
| MYHAT (1652) | 12.9 (2.4) | - | 28.1 (5.4) | 1.8 (29) | 6.9 (114) | 45.2 (746) | 47.7 (788) | 0.2 (4) |
| PATH (2238) | 13.9 (2.8) | 5.5 (123) | 27.1 (4.7) | 27.4 (614) | 9.7 (218) | 37.2 (833) | 53.0 (1187) | - |
| SALSA (1456) | 7.6 (5.4) | - | 29.8 (5.6) | 4.6 (67) | 11.0 (160) | 43.3 (630) | 45.7 (666) | - |
| SPAH (1595) | 2.6 (3.0) | 6.0 (96) | 26.1 (4.6) | 3.2 (51) | 13.2 (211) | 40.9 (653) | 45.8 (731) | - |
| Sydney MAS (905) | 11.5 (3.5) | 1.0 (9) | 27.1 (4.4) | 1.7 (15) | 3.0 (27) | 50.6 (458) | 46.2 (418) | 0.2 (2) |
| Zarademp (3099) | 7.5 (3.9) | 0.7 (23) | 27.0 (4.8) | 0.2 (6) | 13.6 (420) | 22.0 (683) | 64.4 (1996) | - |
| Total (24478) | 9.6 (5.0) | 4.4 (1080) | 26.3 (4.7) | 11.5 (2820) | 10.0 (2452) | 31.8 (7788) | 57.2 (14008) | 0.9 (230) |

*Note.* EAS, Einstein Aging Study; EPIDEMCA, Epidemiology of Dementia in Central Africa; ESPRIT, Etude Santé Psychologique Prévalence Risques et Traitement; FHS, Framingham Heart Study; H70, Gothenburg H70 Birth Cohort Studies; HELIAD, Hellenic Longitudinal Investigation of Aging and Diet; KLOSCAD, Korean Longitudinal Study on Cognitive Aging and Dementia; LEILA75+, Leipzig Longitudinal Study of the Aged; MAAS, Maastricht Ageing Study; MYHAT, Monongahela-Youghiogheny Healthy Aging Team; PATH, Personality and Total Health Through Life Project; SALSA, Sacramento Area Latino Study on Aging; SPAH, São Paulo Ageing and Health Study; Sydney MAS, Sydney Memory and Ageing Study; ZARADEMP, Zaragoza Dementia Depression Project.

Table S8: Clinical characteristics of contributing cohorts

|  | Depression | | Stroke | | Diabetes | | Myocardial infarction | | Hypertension | | Cholesterol | |
| --- | --- | --- | --- | --- | --- | --- | --- | --- | --- | --- | --- | --- |
| Cohort (n) | % (n) | Missing % (n) | % (n) | Missing % (n) | % (n) | Missing % (n) | % (n) | Missing % (n) | % (n) | Missing % (n) | % (n) | Missing % (n) |
| EAS (1284) | 8.2 (105) | 16.1 (207) | 8.0 (103) | 3.0 (39) | 15.9 (204) | 1.6 (21) | 9.0 (115) | 2.8 (36) | 70.9 (910) | 0.3 (4) | 6.5 (84) | 57.9 (743) |
| EPIDEMCA (721) | 39.5 (285) | - | 5.1 (37) | - | 12.2 (88) | 1.5 (11) | - | 100 (721) | 68.7 (495) | 0.6 (4) | 2.6 (19) | 16.9 (122) |
| ESPRIT (1917) | 29.2 (559) | 0.9 (17) | 3.0 (58) | 0.9 (17) | 7.7 (147) | 0.1 (1) | 11.7 (225) | - | 77 (1477) | - | 32.1 (615) | - |
| FHS (1658) | 1.7 (28) | 1.7 (28) | 3.4 (57) | - | 5.4 (89) | 1.6 (27) | 3.9 (65) | 2.3 (38) | 50.8 (842) | 1.6 (26) | 36.2 (601) | 20.2 (335) |
| H70 (576) | 13.5 (78) | - | 14.9 (86) | - | 8.2 (47) | 8.2 (47) | 5.4 (31) | 7.5 (43) | 78.3 (451) | 7.3 (42) | 36.6 (211) | 1.0 (6) |
| HELIAD (954) | 13.5 (129) | - | 7.5 (72) | - | 17.3 (165) | 0.2 (2) | 2.6 (25) | 0.3 (3) | 66.8 (637) | 0.2 (2) | 43.4 (414) | 0.2 (2) |
| KLOSCAD (5139) | 18.9 (972) | 2.6 (135) | 7.1 (366) | 1.1 (55) | 18.6 (956) | 1.5 (78) | 1.9 (97) | 1.1 (59) | 52.1 (2675) | 1.4 (74) | 24.5 (1261) | 1.7 (86) |
| LEILA75+ (851) | 30.8 (262) | 12.5 (106) | 6.2 (53) | - | 22.1 (188) | - | 8.2 (70) | 0.4 (3) | 79 (672) | 4.3 (37) | - | - |
| MAAS (433) | 23.8 (103) | 3.2 (14) | - | 100 (433) | 7.9 (34) | - | 5.5 (24) | - | 56.8 (246) | 1.2 (5) | 12.2 (53) | - |
| MYHAT (1652) | 11 (181) | 0.1 (2) | 4.3 (71) | - | 21.6 (357) | - | 14.4 (238) | 0.1 (1) | 82.1 (1357) | - | 68.5 (1131) | - |
| PATH (2238) | 5.1 (115) | 0.2 (5) | 3.9 (88) | - | 7.1 (160) | - | 14.8 (332) | 0.1 (3) | 65.6 (1469) | 0.04 (1) | 23.3 (521) | - |
| SALSA (1456) | 24 (350) | 0.8 (11) | 8.4 (123) | - | 31.5 (459) | - | 8.2 (120) | - | 62.9 (916) | - | 42.7 (622) | - |
| SPAH (1595) | 4.1 (65) | 0.3 (4) | 7.4 (118) | - | 20.9 (333) | 3.9 (62) | - | 100 (1595) | 78.7 (1256) | - | 31.8 (508) | 4.3 (69) |
| Sydney MAS (905) | 6.5 (59) | 0.2 (2) | 3.9 (35) | 0.9 (8) | 11.3 (102) | 0.6 (5) | 10.5 (95) | 0.4 (4) | 81.4 (737) | - | 64.2 (581) | 0.1 (1) |
| Zarademp (3099) | 16.3 (506) | 4.4 (136) | 2.8 (86) | 2.8 (86) | 12.3 (382) | 0.8 (26) | 2.8 (86) | 1.5 (46) | 68.1 (2111) | 0.2 (5) | 6.1 (190) |  |
| Total (24478) | 15.5 (3797) | 2.7 (667) | 5.5 (1353) | 2.6 (638) | 15.2 (3711) | 1.1 (280) | 6.2 (1523) | 10.4 (2552) | 66.4 (16251) | 0.8 (200) | 27.8 (6811) | 9.0 (2215) |

*Note.* EAS, Einstein Aging Study; EPIDEMCA, Epidemiology of Dementia in Central Africa; ESPRIT, Etude Santé Psychologique Prévalence Risques et Traitement; FHS, Framingham Heart Study; H70, Gothenburg H70 Birth Cohort Studies; HELIAD, Hellenic Longitudinal Investigation of Aging and Diet; KLOSCAD, Korean Longitudinal Study on Cognitive Aging and Dementia; LEILA75+, Leipzig Longitudinal Study of the Aged; MAAS, Maastricht Ageing Study; MYHAT, Monongahela-Youghiogheny Healthy Aging Team; PATH, Personality and Total Health Through Life Project; SALSA, Sacramento Area Latino Study on Aging; SPAH, São Paulo Ageing and Health Study; Sydney MAS, Sydney Memory and Ageing Study; ZARADEMP, Zaragoza Dementia Depression Project.

Table S9. Comparison of baseline demographic and clinical characteristics of the participants included and excluded from the analyses

|  | Included (n=24,478) | | Excluded  (n=7,885) | | *p* |
| --- | --- | --- | --- | --- | --- |
|  | Mean | SD | Mean | SD |  |
| Age | 71.8 | 7.5 | 75.6 | 8.5 | <.001 |
| Education | 9.6 | 5.0 | 8.1 | 5.1 | <.001 |
| BMI | 26.3 | 4.7 | 26.2 | 5.2 | .026 |
|  | N | % | N | % |  |
| Female | 14260 | 58.3 | 5614 | 62.1 | <.001 |
| Former smoker | 7788 | 31.8 | 2293 | 25.3 | <.001 |
| Current smoker | 2452 | 10.0 | 890 | 9.8 |  |
| Alcohol consumption |  |  |  |  |  |
| Abstainer | 11209 | 45.8 | 4606 | 58.4 | <.001 |
| Former drinker | 4091 | 16.7 | 1707 | 21.6 |  |
| <1.3g/day | 3255 | 13.3 | 891 | 11.3 |  |
| 1.3-24.9g/day | 7626 | 31.2 | 1841 | 23.4 |  |
| 25-44.9g/day | 1581 | 6.5 | 329 | 4.2 |  |
| >45g/day | 807 | 3.3 | 217 | 2.8 |  |
| Clinical characteristics |  |  |  |  |  |
| Depression | 3797 | 15.9 | 1692 | 22.5 | <.001 |
| Stroke | 1353 | 5.7 | 892 | 10.6 | <.001 |
| Diabetes | 3711 | 15.3 | 1505 | 18.0 | <.001 |
| Hypertension | 16251 | 66.9 | 5574 | 65.9 | .094 |
| Myocardial Infarction | 1523 | 6.9 | 581 | 7.7 | .019 |
| High cholesterol | 6811 | 30.6 | 1646 | 23.4 | <.001 |

| Baseline drinking category | Baseline dementia present (n=1,522) | | Baseline dementia absent (n=31,740) | | *p* |
| --- | --- | --- | --- | --- | --- |
|  | N | % | N | % |  |
| Abstainer | 14,885 | 48.0 | 940 | 68.5 | <.001 |
| Former drinker | 5398 | 17.5 | 368 | 26.8 |  |
| <1.3g/day | 3962 | 12.8 | 171 | 12.5 |  |
| 1.3-24.9g/day | 9250 | 29.9 | 206 | 15.0 |  |
| 25-44.9g/day | 1877 | 6.1 | 33 | 2.4 |  |
| >45g/day | 1001 | 3.2 | 23 | 1.7 |  |
|  | | | | | |

Table S10. Comparison of baseline alcohol use characteristics of participants with and without baseline dementia diagnosis

Table S11. Relationships between demographic and clinical covariates and dementia risk in the combined sample (n=24,478) ^a^

| Covariate | Hazard Ratio (95% Confidence Limits) |
| --- | --- |
| Age | 1.15 (1.14, 1.16) |
| Sex (Female) | 1.14 (1.04, 1.24) |
| Smoking (Former Smoker) | 0.85 (0.77, 0.93) |
| Smoking (Current Smoker) | 0.79 (0.68, 0.92) |
| Body Mass Index | 0.97 (0.96, 0.98) |
| Education | 0.91 (0.90, 0.92) |
| Depression | 1.82 (1.62, 2.05) |
| Stroke | 2.15 (1.85, 2.51) |
| Diabetes | 1.42 (1.26, 1.60) |
| Hypertension | 1.24 (1.13, 1.36) |
| Myocardial Infarction | 1.29 (1.10, 1.52) |
| High cholesterol | 0.93 (0.84, 1.03) |

^a^ Separate models were implemented for each covariate independently and included a random effect for study and inverse probability of censoring weights.

Table S12. Combined sample hazard ratios with lifetime abstainers and former drinkers separated

|  | Main model ^a^ | Fully adjusted model ^b^ | Competing risk model ^c^ |
| --- | --- | --- | --- |
| Combined sample | n=20,187 | n=18,592 | n=16,354 |
| Lifetime abstainer | ref | ref | ref |
| Former drinkers | 0.98 (0.81, 1.18) | **0.90 (0.73, 1.10)** | 0.91 (0.72, 1.14) |
| ≤1.3g/day | **0.71 (0.59, 0.85)** | **0.71 (0.57, 0.88)** | 0.70 (0.45, 1.09) |
| 1.3-24.9g/day | **0.69 (0.59, 0.81)** | **0.72 (0.60, 0.85)** | **0.75 (0.57, 0.99)** |
| 25-44.9g/day | **0.49 (0.36, 0.65)** | **0.56 (0.42, 0.75)** | **0.50 (0.38, 0.67)** |
| ≥45g/day | 0.90 (0.62, 1.29) | 0.77 (0.51, 1.15) | 0.87 (0.53, 1.43) |
| Male sample | n=8,431 | - | - |
| Lifetime abstainer | ref |  |  |
| Former drinker | 1.13 (0.82, 1.57) |  |  |
| ≤1.3g/day | 0.86 (0.59, 1.26) |  |  |
| 1.3-24.9g/day | 0.77 (0.58, 1.04) |  |  |
| 25-44.9g/day | **0.58 (0.39, 0.85)** |  |  |
| ≥45g/day | 0.97 (0.63, 1.50) |  |  |
| Female sample | n=11,756 | - | - |
| Lifetime abstainer | ref |  |  |
| Former drinker | 0.92 (0.71, 1.17) |  |  |
| ≤1.3g/day | **0.65 (0.52, 0.81)** |  |  |
| 1.3-24.9g/day | **0.68 (0.56, 0.83)** |  |  |
| 25-44.9g/day | **0.38 (0.22, 0.67)** |  |  |
| ≥45g/day | 0.59 (0.16, 2.18) |  |  |

^a^ Model included 11 cohorts and adjusted for age, sex smoking status and random effect of study; ^b^ Model included 10 cohorts and adjusted for age, sex, smoking status, education, BMI, depression stroke, diabetes, myocardial infarction, hypertension, high cholesterol and random effect for study; ^c^ Model included 9 cohorts, adjusted for age, sex, smoking status and study as a cluster variable, and accounted for competing risk of death

Figure S1. Dose response relationship between usual alcohol use (grams/day; implemented using the regression dilution ratio) and dementia including lifetime abstainers (reference group) and current drinkers


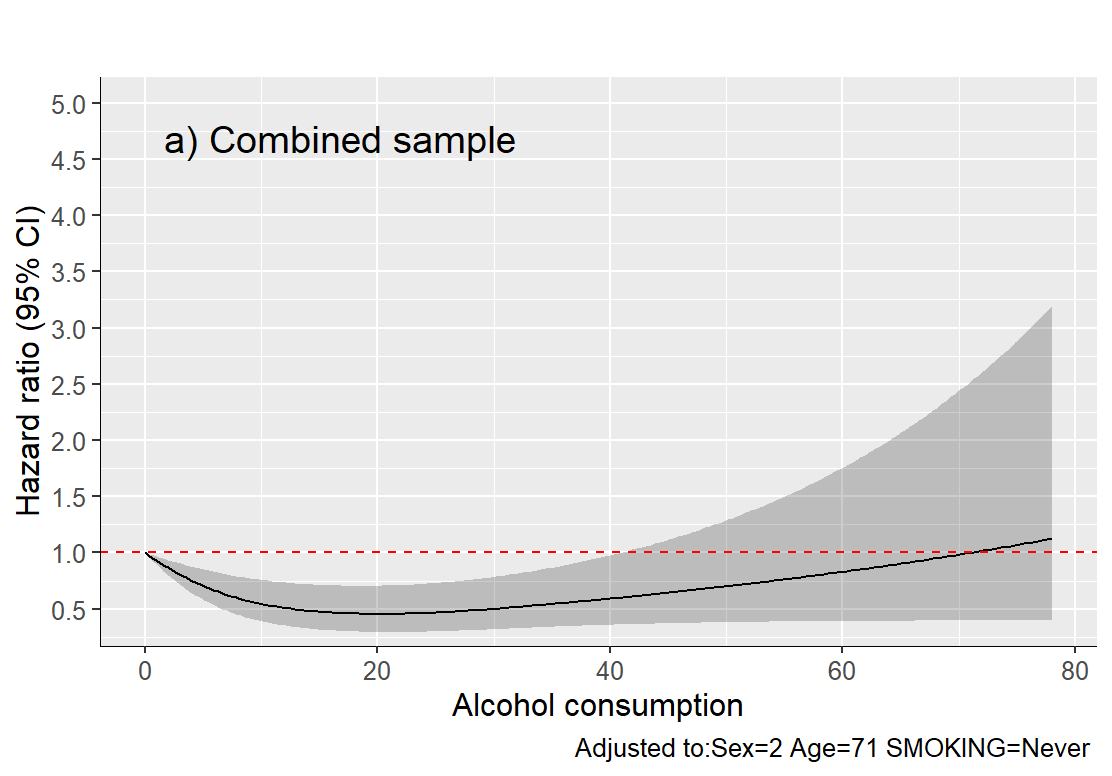

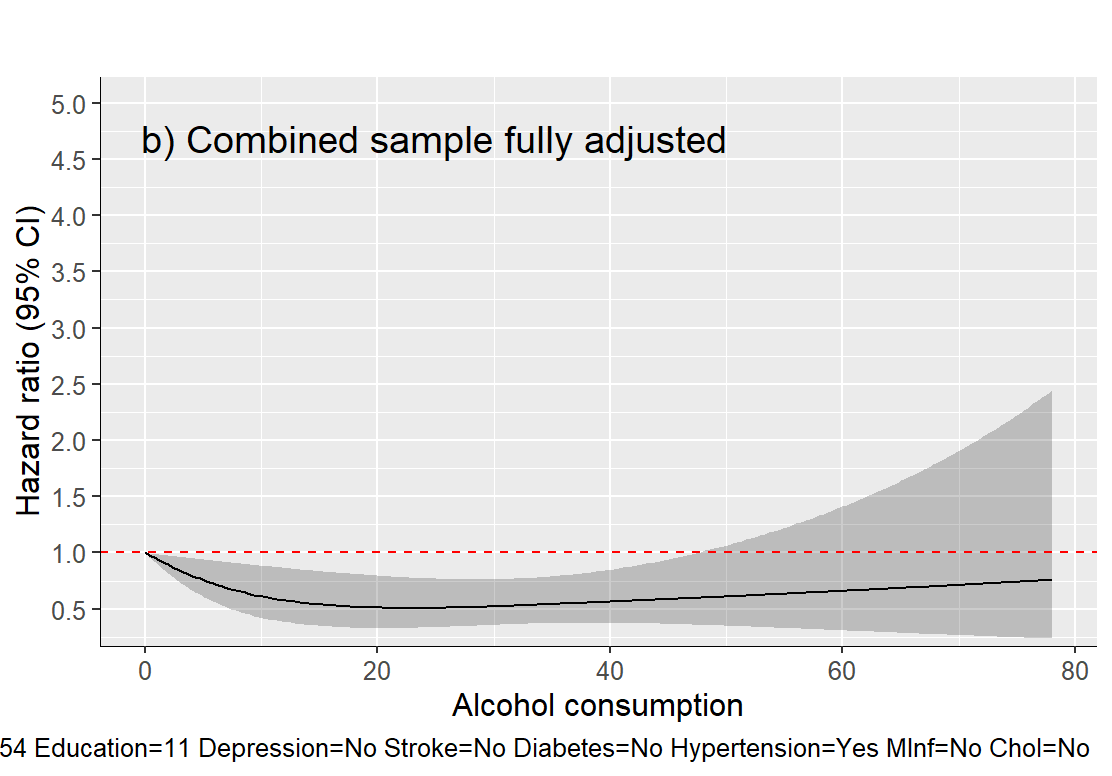

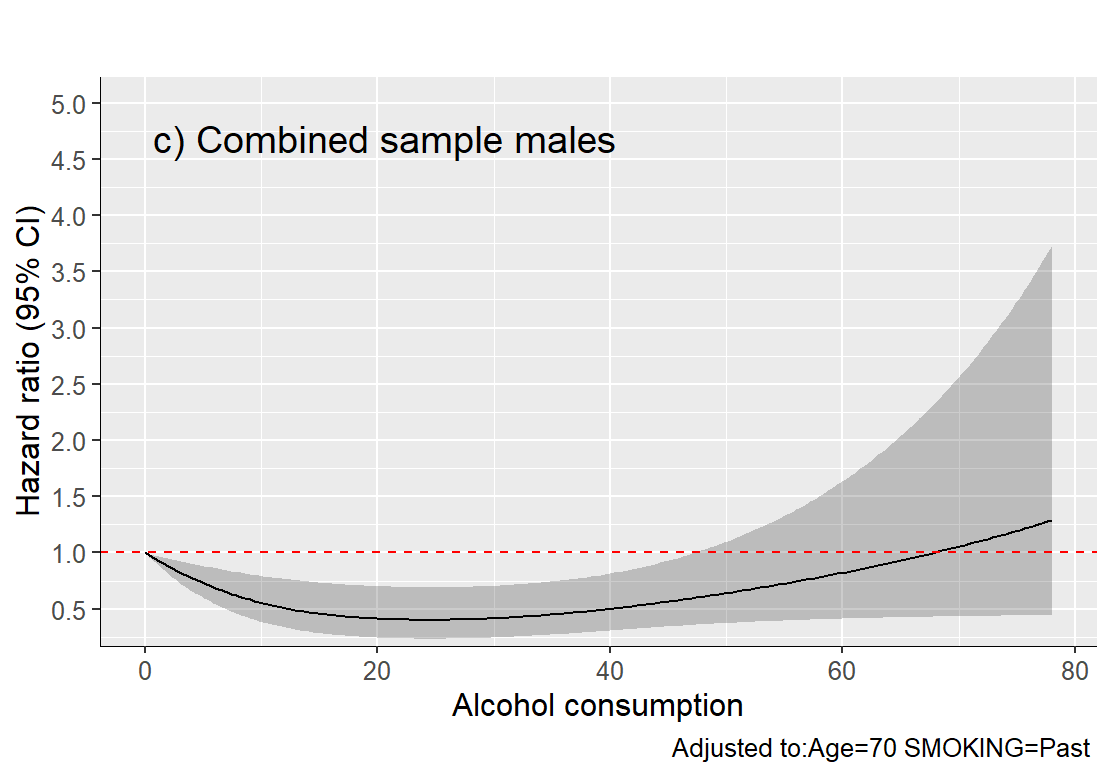

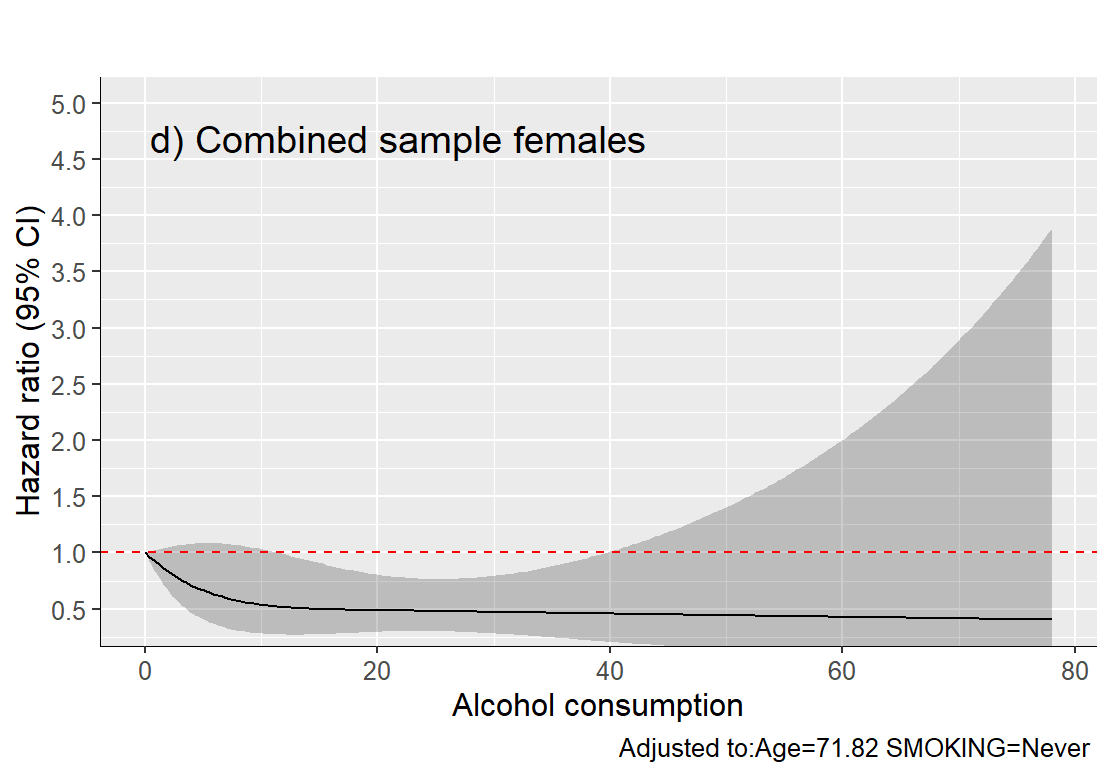
Note: a) Model included 11 cohorts with former drinkers excluded (n=17,964) and adjusted for age, smoking status and random effect of study; b) Model included 10 cohorts with former drinkers excluded (n=15,979) and adjusted for age, sex, smoking status, education, BMI, depression stroke, diabetes, myocardial infarction, hypertension, high cholesterol and random effect for study; c) Model included males 11 cohorts with former drinkers excluded (n=7,216) and adjusted for age, smoking status and random effect of study; d) Model included females in 11 cohorts with former drinkers excluded (n=10,748) and adjusted for age, smoking status and random effect of study.

Figure S2. Dose response relationship between usual alcohol use (grams/day; implemented using the regression dilution ratio) and dementia among current drinkers


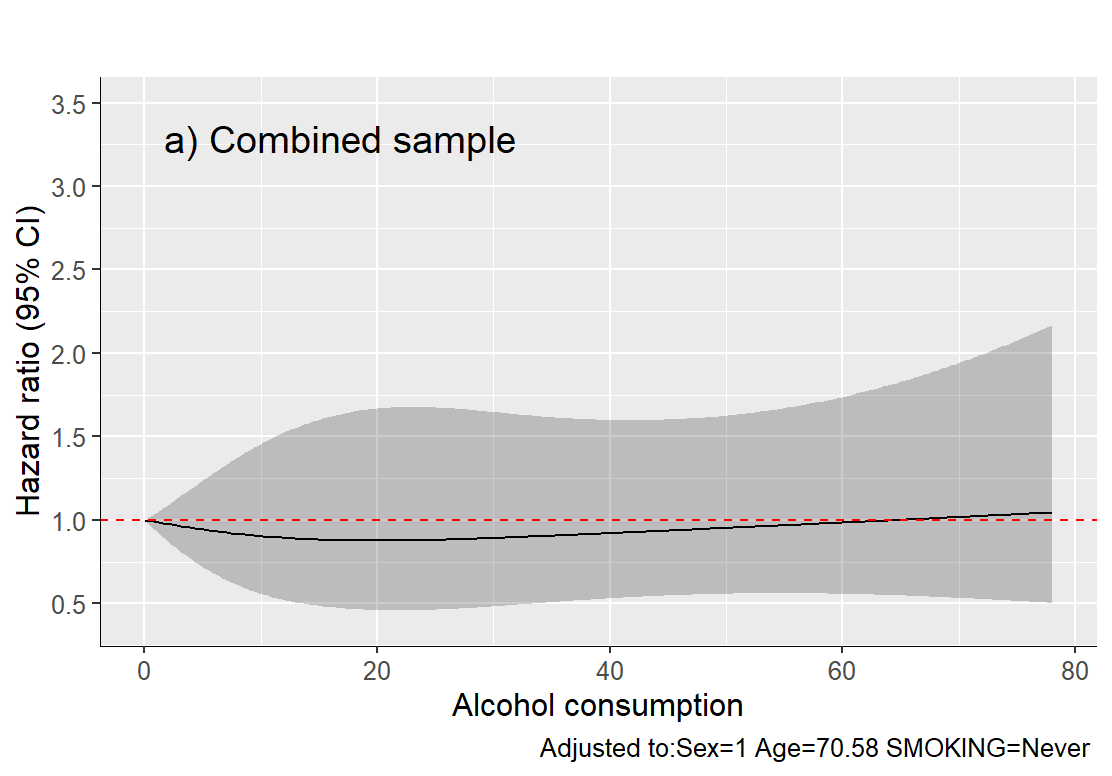

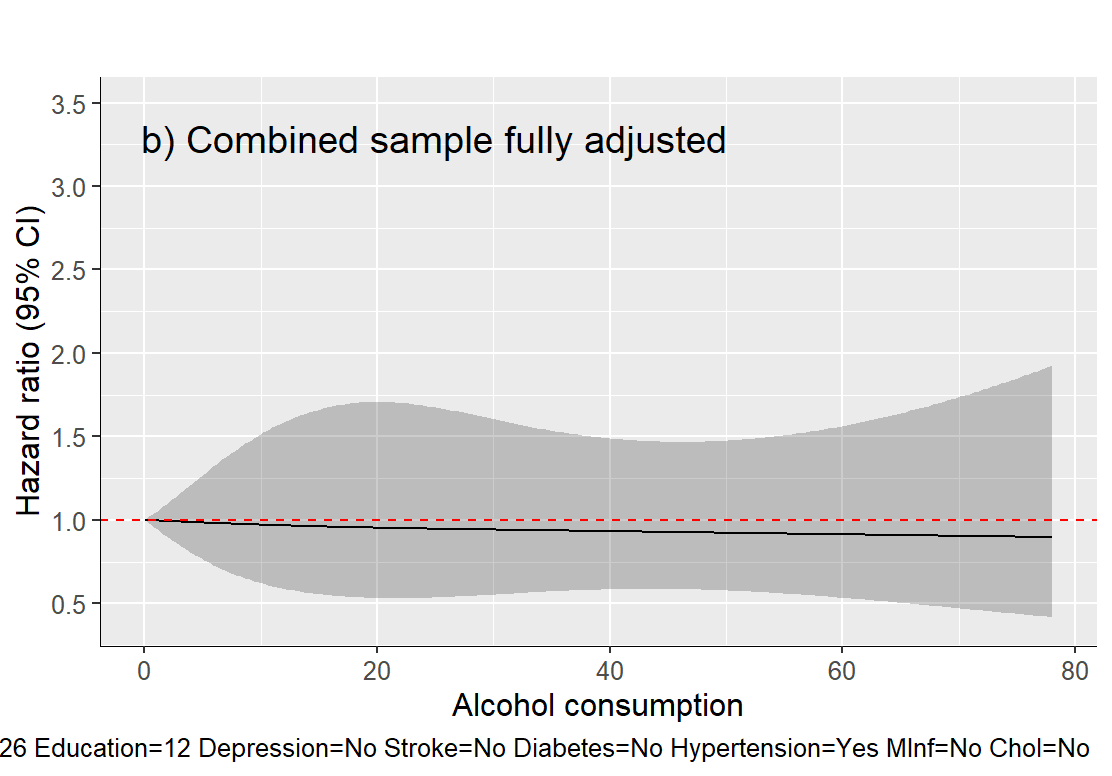

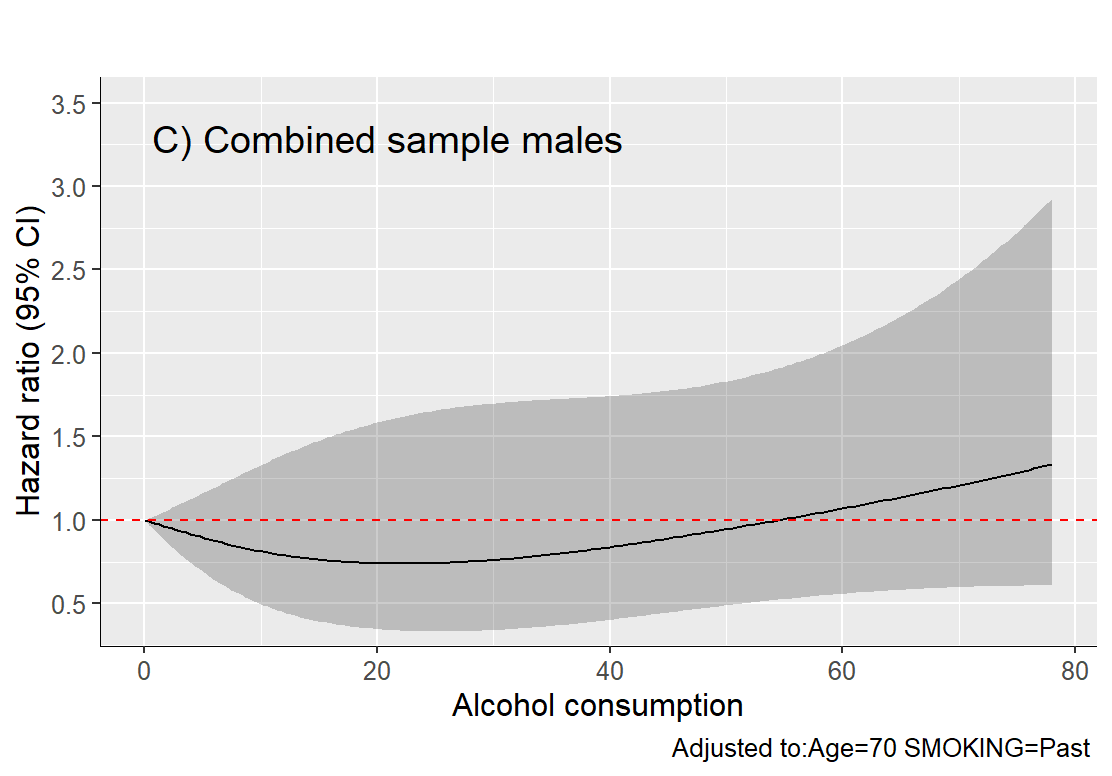

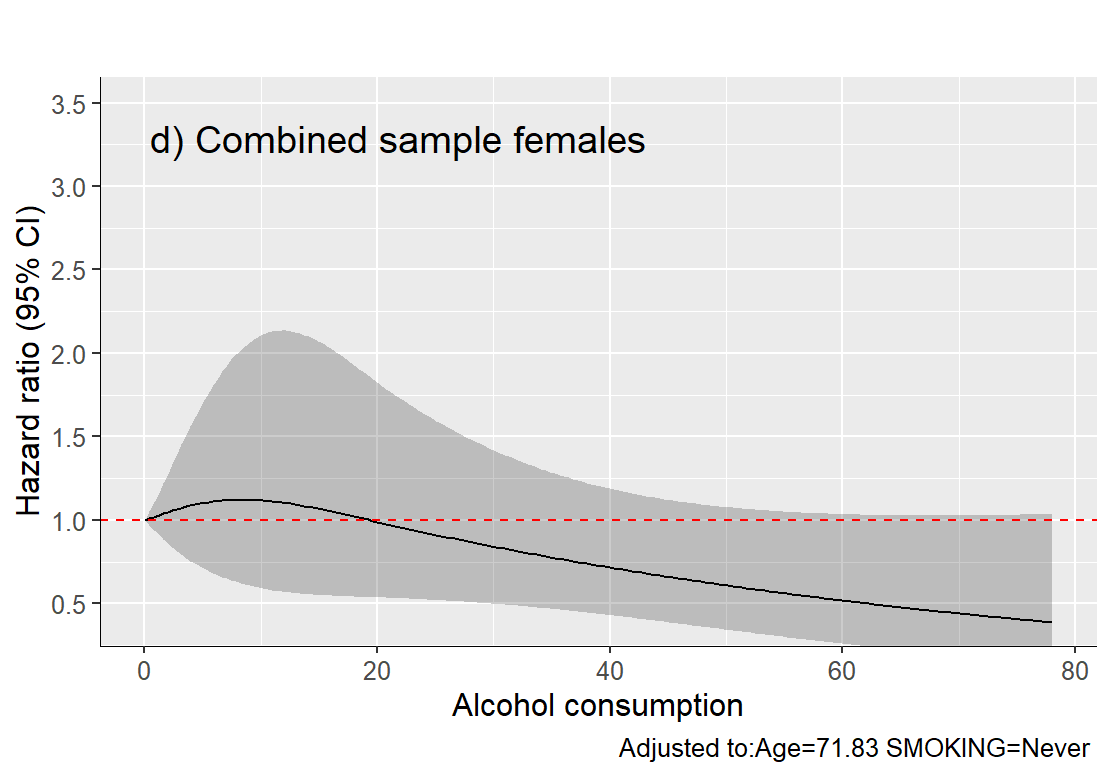


Note: a) Model included current drinkers from 15 cohorts (n=13,335) and adjusted for age, smoking status and random effect of study; b) Model included current drinkers from 11 cohorts (n=11,722) and adjusted for age, sex, smoking status, education, BMI, depression stroke, diabetes, myocardial infarction, hypertension, high cholesterol and random effect for study; c) Model included male current drinkers from 15 cohorts (n=7,063) and adjusted for age, smoking status and random effect of study; d) Model included female current drinkers in 15 cohorts with former drinkers excluded (n=6,272) and adjusted for age, smoking status and random effect of study.

Figure S3. Sensitivity analysis excluding participants reporting stroke at baseline


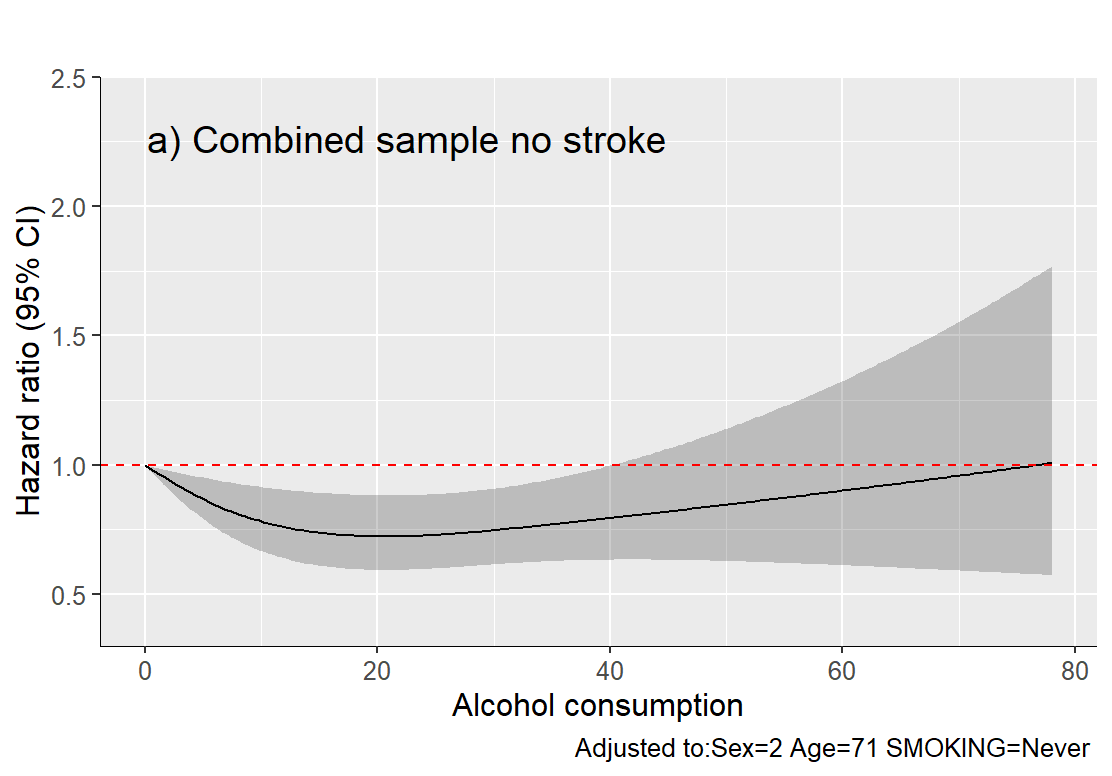

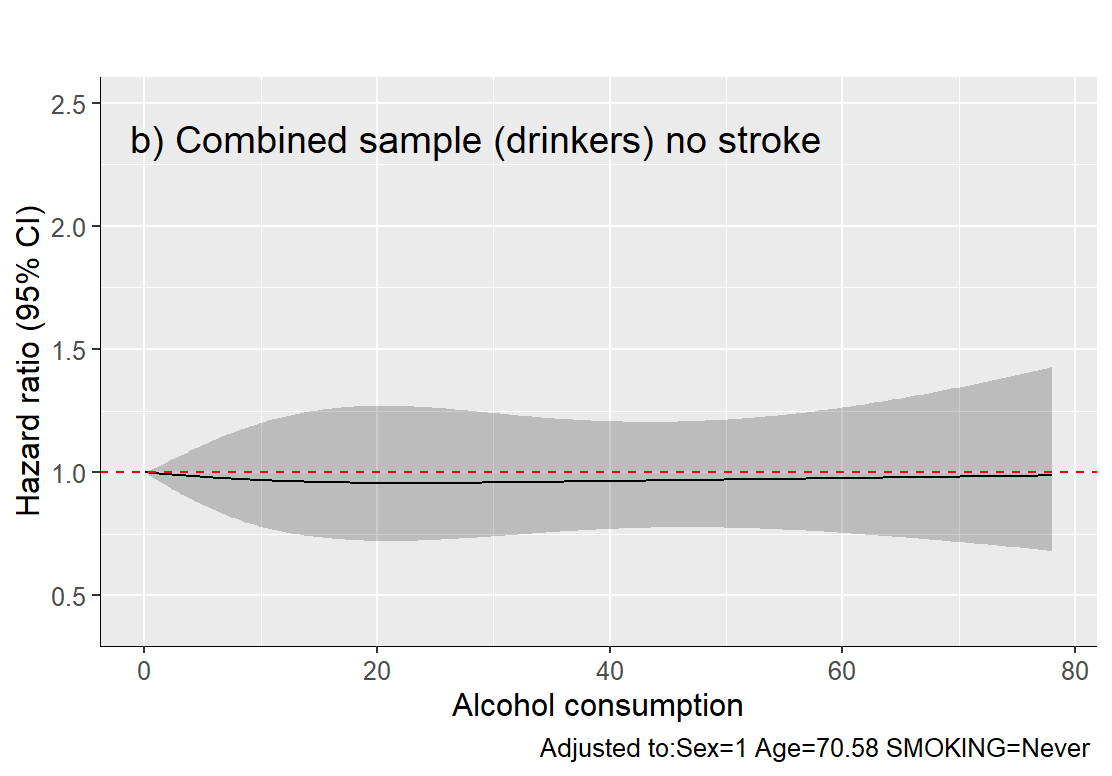


Note: a) Model included 11 cohorts with former drinkers excluded (n=17,016) and adjusted for age, smoking status and random effect of study; b) Model included current drinkers from 15 cohorts (n=12,343) and adjusted for age, smoking status and random effect of study

Figure S4. Sub-group analyses by continent including lifetime abstainers (reference group) and current drinkers


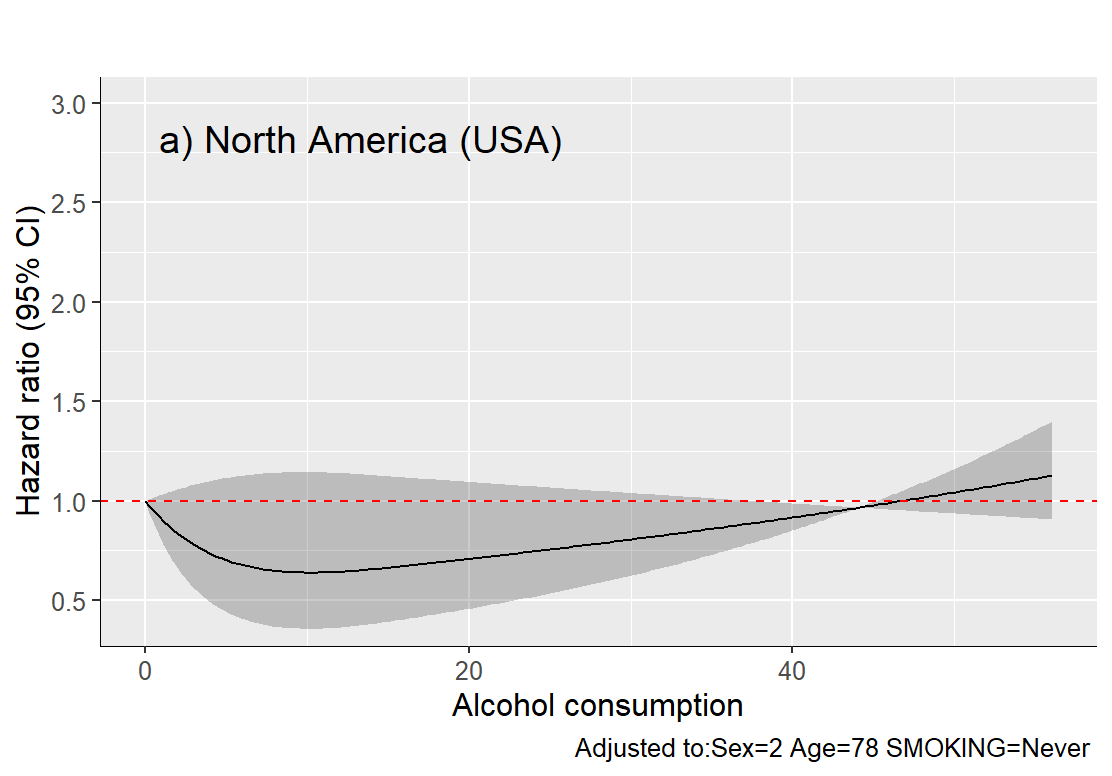

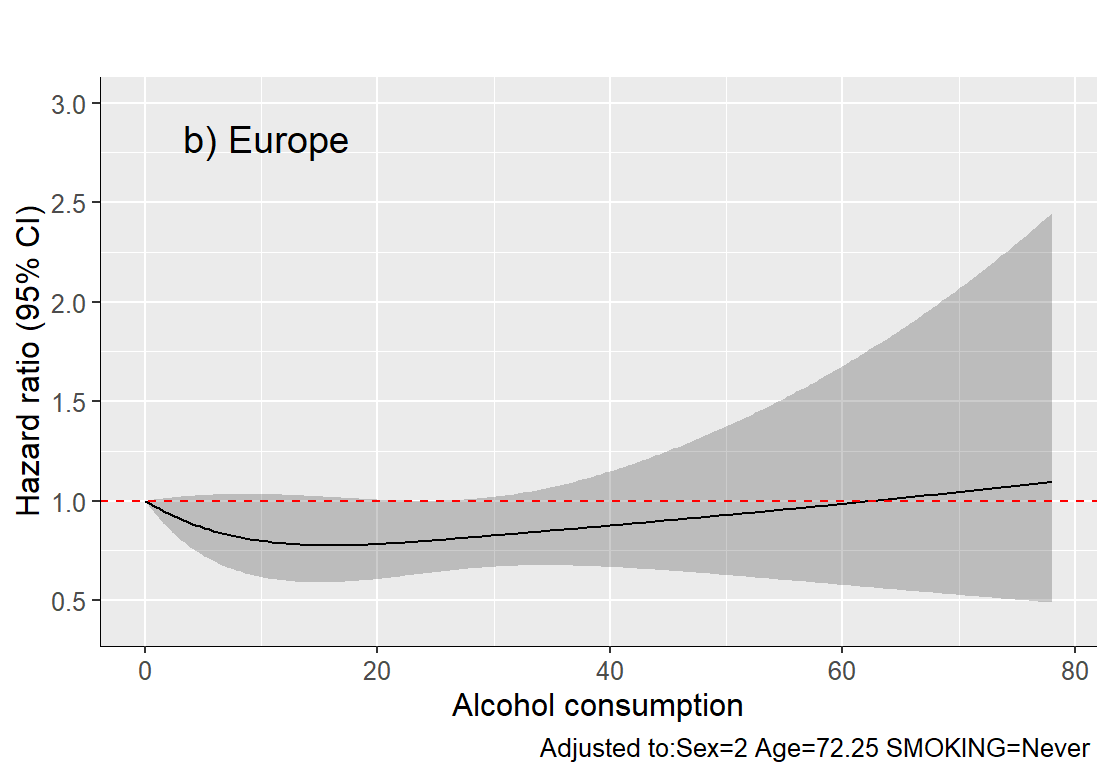


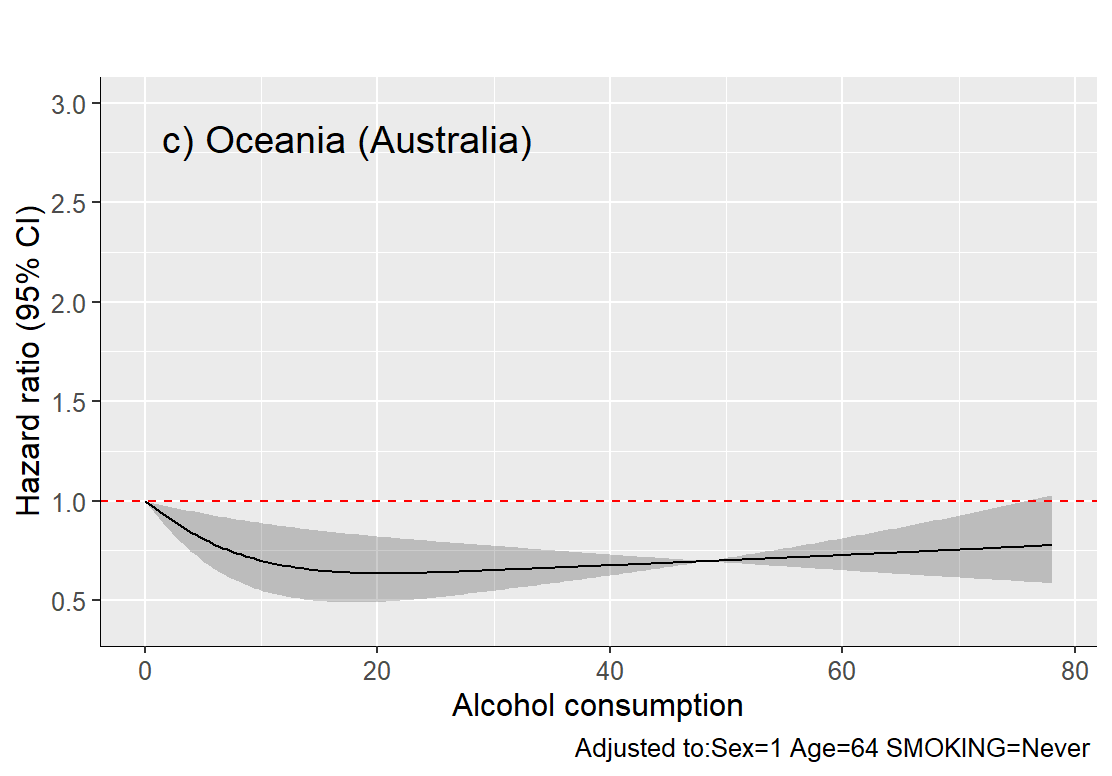

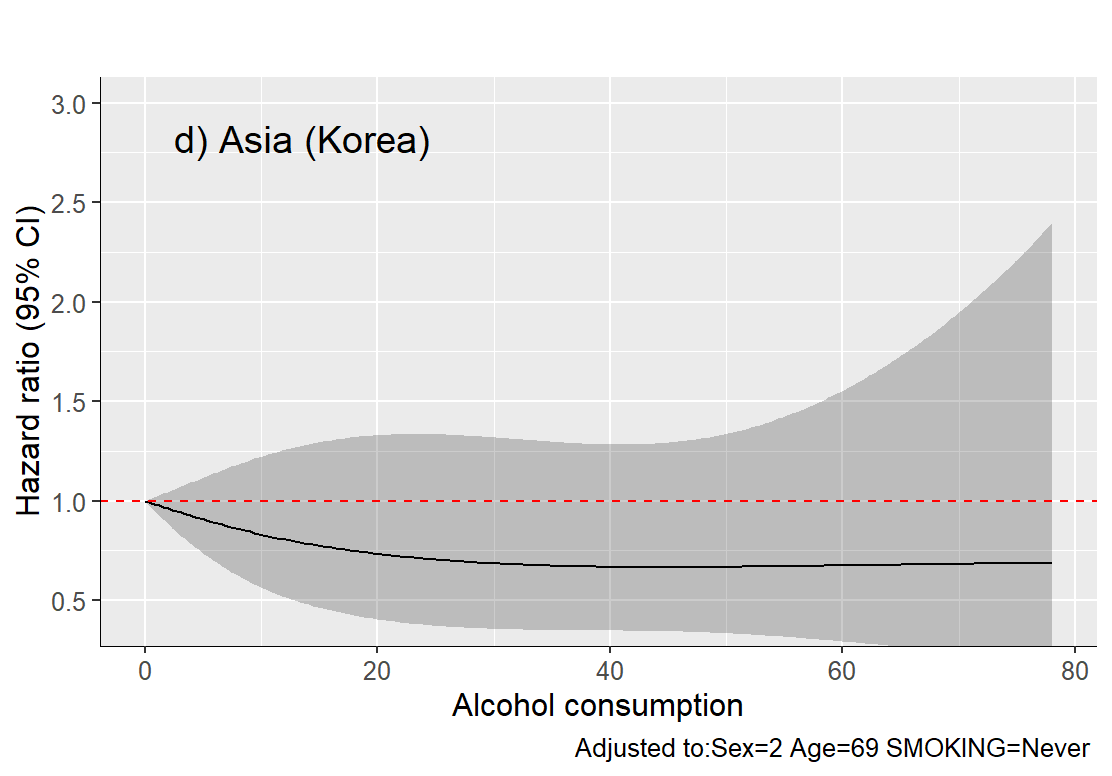
 Note: a) Model included 2 cohorts with former drinkers excluded (n=2,380) and adjusted for age, smoking status and random effect of study; b) Model included 5 cohorts with former drinkers excluded (n=6,735) and adjusted for age, smoking status and random effect of study; c) Model included 2 cohorts with former drinkers excluded (n=2,898) and adjusted for age, smoking status and random effect of study; d) Model included 1 cohort with former drinkers excluded (n=4,737) and adjusted for age, smoking status and random effect of study

Figure S5. Sub-group analyses by continent including current drinkers


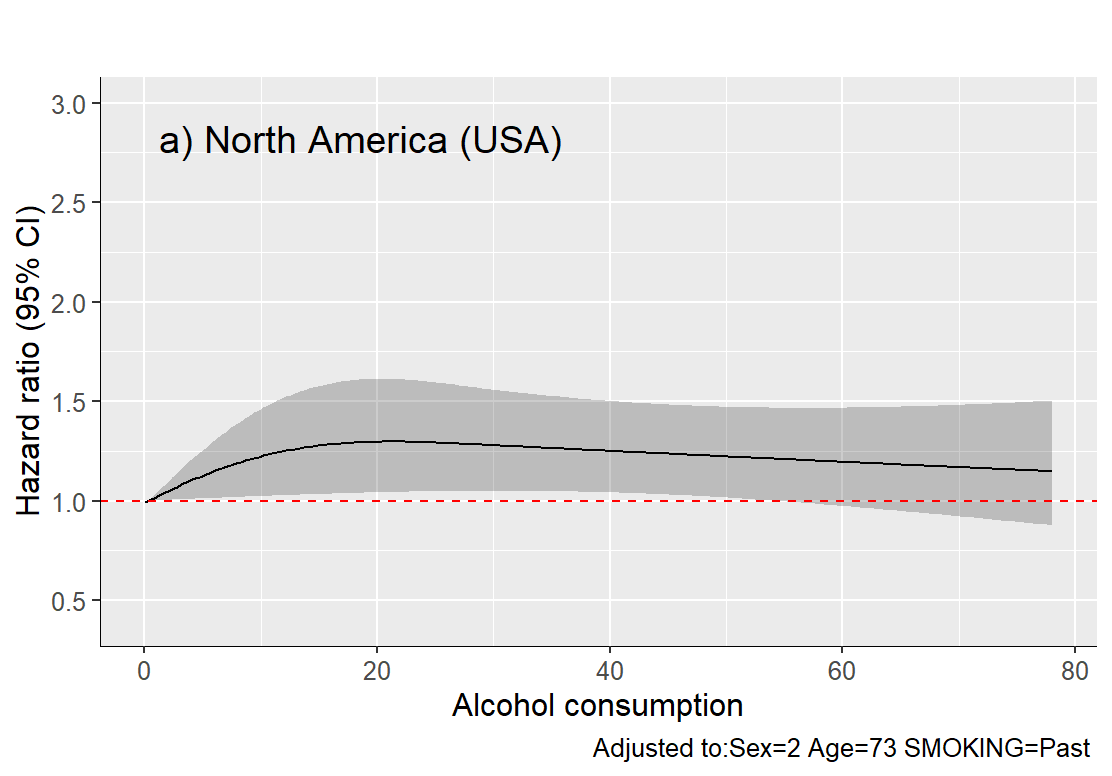

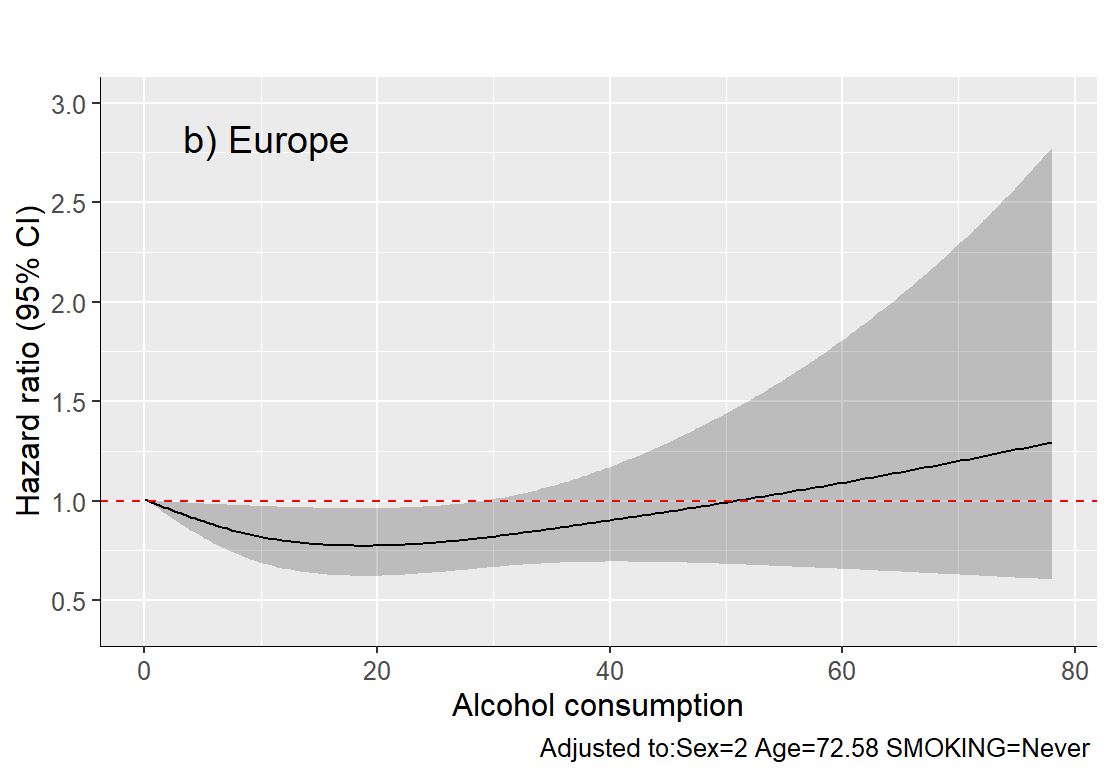

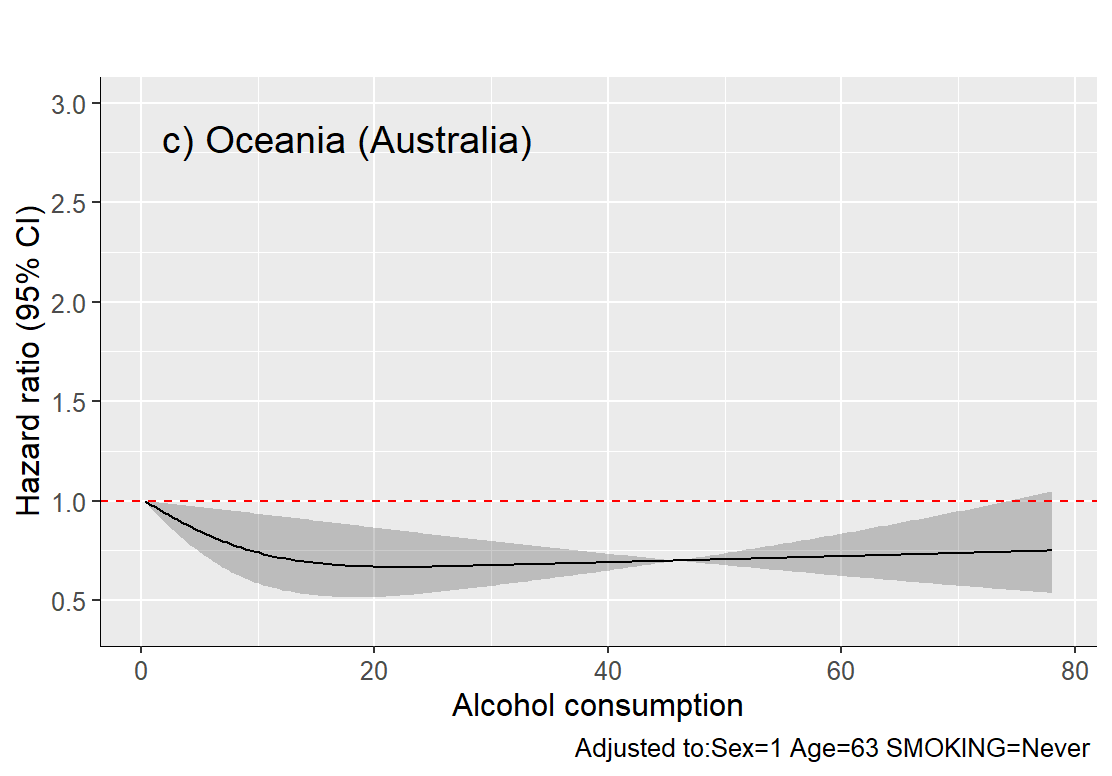

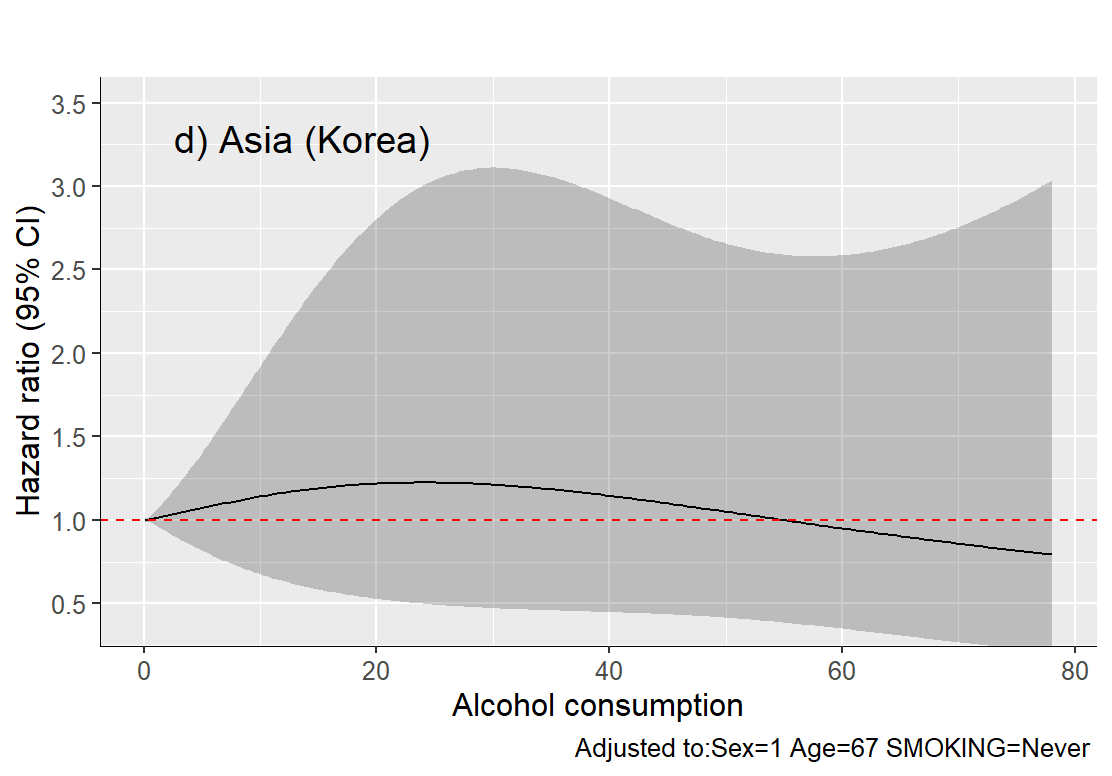

Note: a) Model included current drinkers from 4 cohorts (n=3,877) and adjusted for age, smoking status and random effect of study; b) Model included current drinkers from 6 cohorts (n=4,527) and adjusted for age, smoking status and random effect of study; c) Model included current drinkers from 2 cohorts (n=2,733) and adjusted for age, smoking status and random effect of study; d) Model included current drinkers from 1 cohort (n=1,664) and adjusted for age, smoking status and random effect of study

**Further detail on multiple imputation and censoring weights**

Participants were excluded from the analysis if they were diagnosed with dementia at baseline. Those without data on baseline alcohol consumption, sex and baseline dementia status were also excluded from the analysis. Then, multiple imputation of other individual-level covariates (BMI, years of education, smoking, depression, stroke, diabetes, myocardial infarction, hypertension and high cholesterol) was performed. The *mice* package [1] was used to generate twenty imputed datasets for each study with a maxit of 20. Predictors for each covariate included all other covariates present in a given study as well as grams of ethanol per day at baseline (rather than alcohol categories). The imputation code was adapted from [2]. The Rhat.mice function [2] from the *miceadds* package [3] was used to check convergence, which was achieved.

Inverse probability of censoring weights (IPCWs) were calculated to account for differential attrition. While IPCWs are often calculated at each timepoint in cohorts with multiple waves (e.g. in marginal structural models), in this study we were interested in only two censoring outcomes: having any follow-up (these participants therefore provided some outcome data), or having no follow-up (these participants had to be excluded from analyses). ICPWs represent the inverse probability of a participant realising the censoring outcome they did, given their observed covariates. Incorporating these weights allows the sample included in the final Cox model to be more representative of the original sample present at baseline, as remaining individuals whose covariates are more typical of those lost to follow-up are up-weighted.

Prior to removal of those without follow-up, the *WeightIt* package [4] was used to generate weights for each individual in *each of the imputed datasets*. We used stabilised IPCWs generated with the CBPS method [5]. Depending on the availability of covariates, variations of the following code was used for each study:

IPCW_form <-"NoFU ~ Grams_Day.1 + SMOKING + Sex + BMI + Education + Age + Stroke + Depression + Hypertension + Chol + MInf + Diabetes"

IPCW_weights_EAS <- with(impEAS,

weightit(as.formula(IPCW_form),

method="cbps",

over=FALSE,

stabilize=TRUE))

“Love” plots made with the *cobalt* package [6] demonstrated that balance was greatly improved in each study (see Figure S1).

| EAS 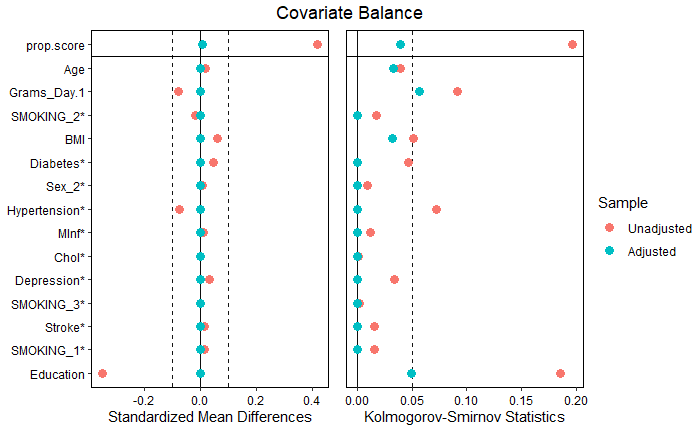 | EPIDEMCA  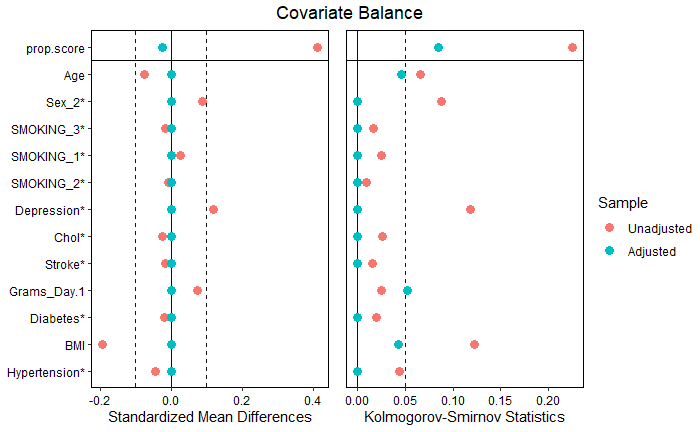 | ESPRIT  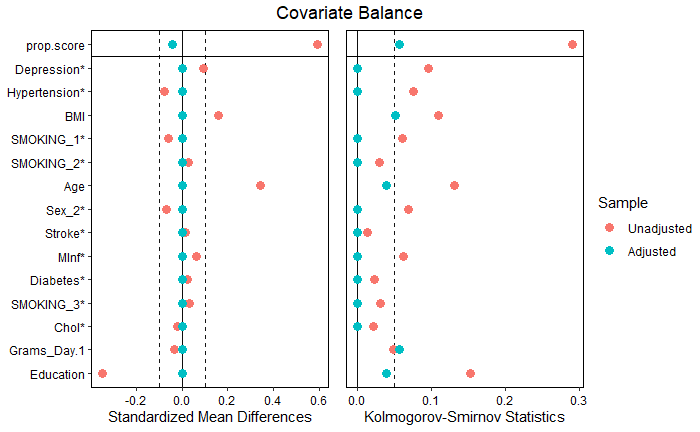 |
| --- | --- | --- |
| FHS  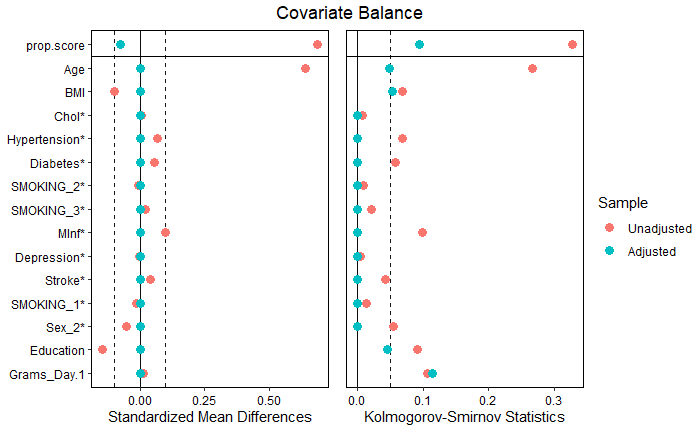 | H70  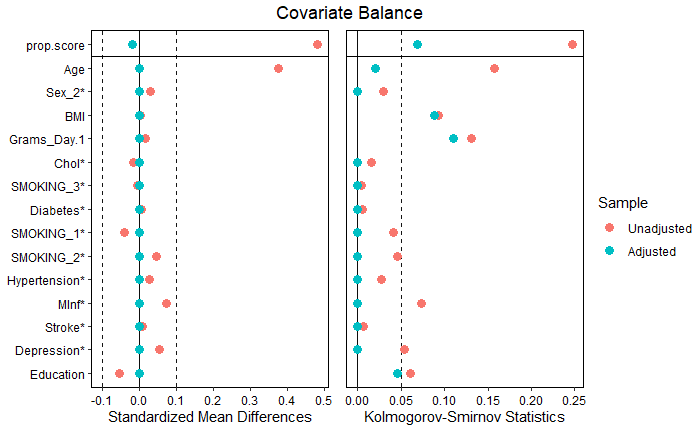 | HELIAD  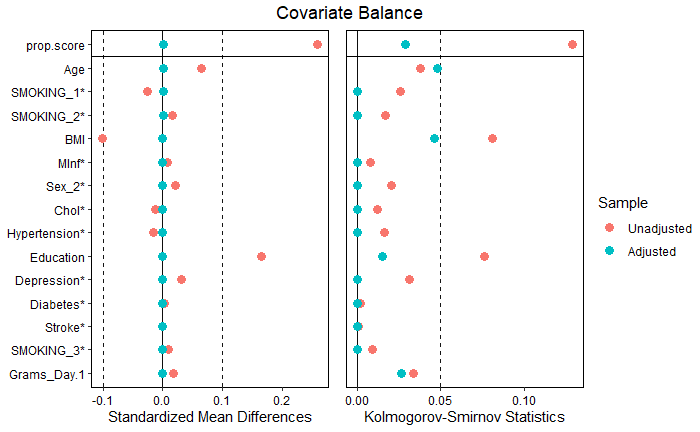 |
| KLOSCAD  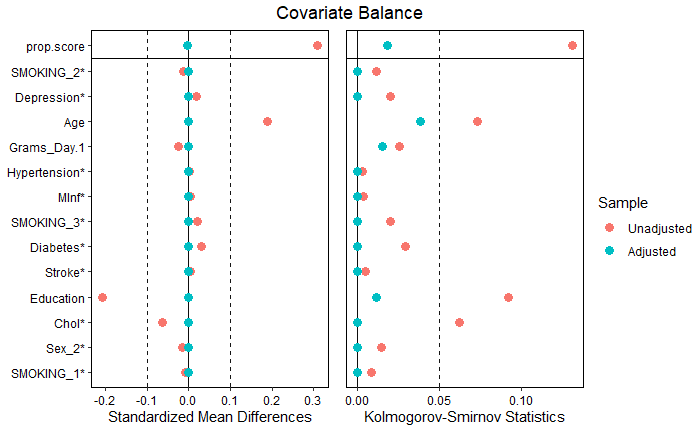 | LEILA 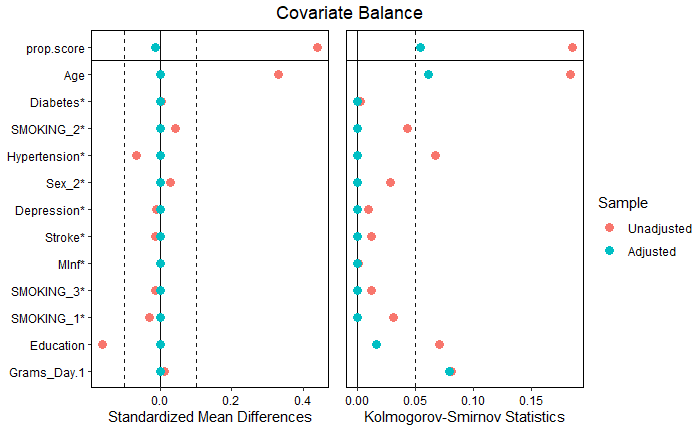 | MAAS  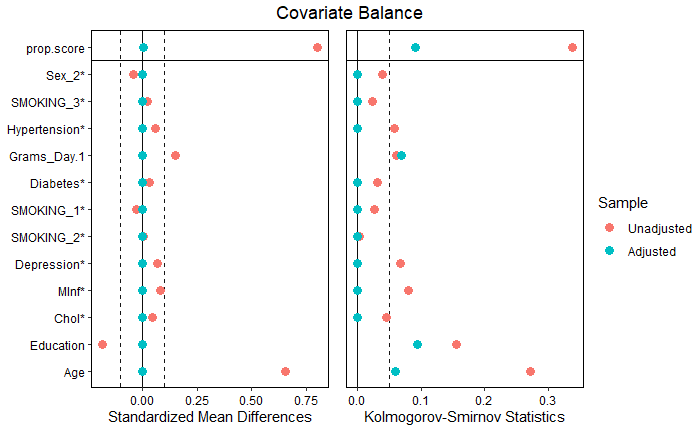 |
| MAS  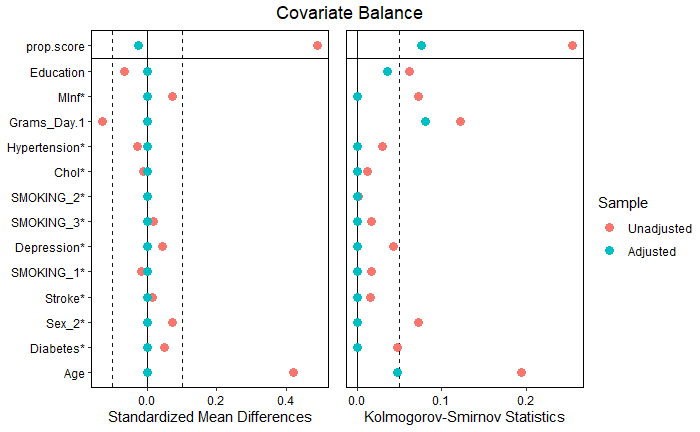 | MYHAT  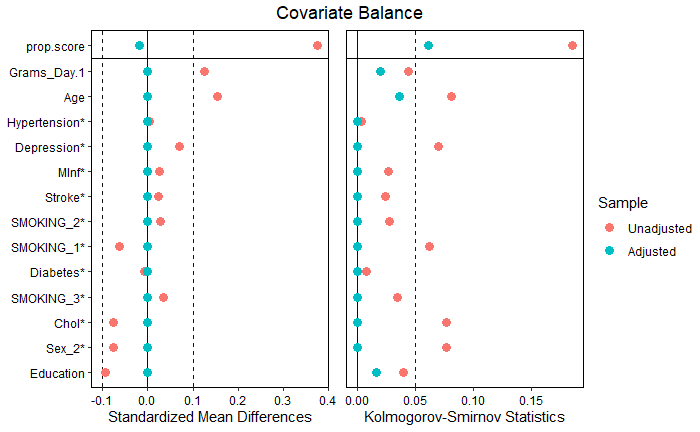 | PATH 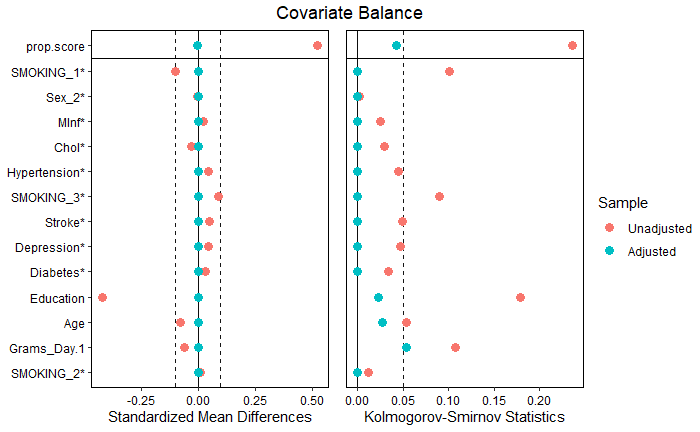 |
| SALSA  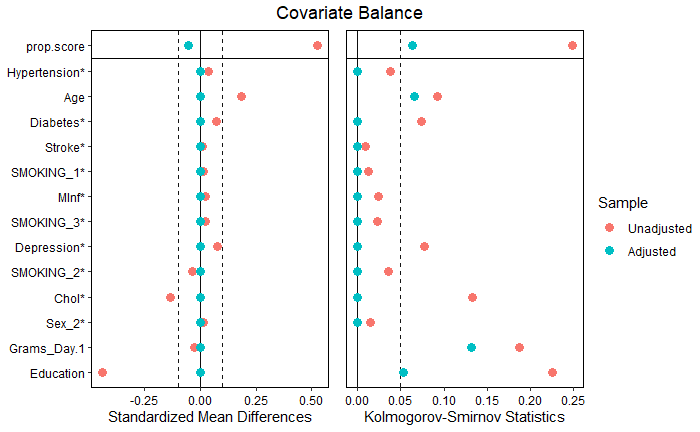 | SPAH 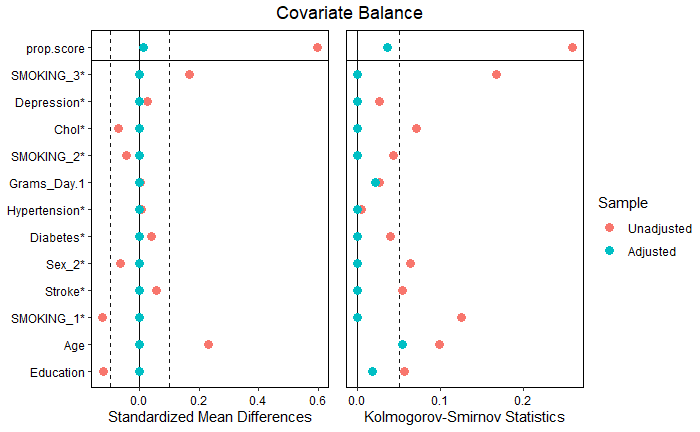 | ZARADEMP  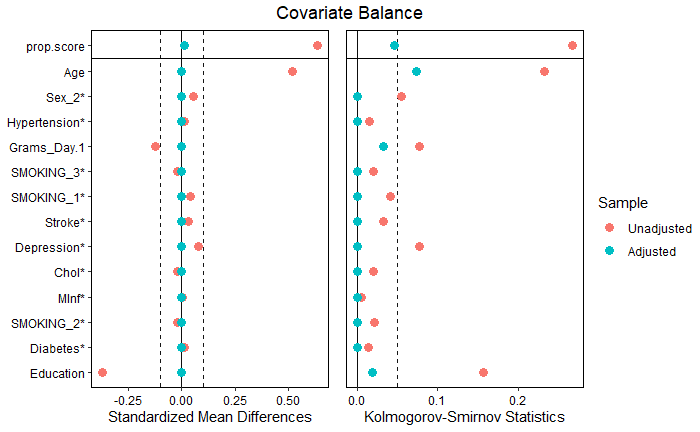 |

Figure S1. Love plots of covariate balance for each cohort.

*Note*. Plots depict covariate balance between censored and uncensored individuals before and after inverse probability of censoring weighting. Plots were generated using the first of each cohort’s twenty imputed datasets. Mean differences for categorical variables (denoted by a tailing asterisk) are raw. EAS, Einstein Aging Study; EPIDEMCA, Epidemiology of Dementia in Central Africa; ESPRIT, Etude Santé Psychologique Prévalence Risques et Traitement; FHS, Framingham Heart Study; H70, Gothenburg H70 Birth Cohort Studies; HELIAD, Hellenic Longitudinal Investigation of Aging and Diet; KLOSCAD, Korean Longitudinal Study on Cognitive Aging and Dementia; LEILA75+, Leipzig Longitudinal Study of the Aged; MAAS, Maastricht Ageing Study; MYHAT, Monongahela-Youghiogheny Healthy Aging Team; PATH, Personality and Total Health Through Life Project; SALSA, Sacramento Area Latino Study on Aging; SPAH, São Paulo Ageing and Health Study; Sydney MAS, Sydney Memory and Ageing Study; ZARADEMP, Zaragoza Dementia Depression Project.

**References**

1. Buuren, S.v. and K. Groothuis-Oudshoorn, *mice: Multivariate imputation by chained equations in R.* Journal of statistical software, 2010: p. 1-68.

2. Yoshida, K. *mice: Excluding variables from imputation*. 2016; Available from: <https://rpubs.com/kaz_yos/mice-exclude>.

3. Robitzsch, A., et al., *Package ‘miceadds’.* R Package: Madison, WI, USA, 2017.

4. Greifer, N. and M.N. Greifer, *Package ‘WeightIt’*. 2019.

5. Imai, K. and M. Ratkovic, *Covariate balancing propensity score.* Journal of the Royal Statistical Society: Series B: Statistical Methodology, 2014: p. 243-263.

6. Greifer, N., *Covariate balance tables and plots: a guide to the cobalt package.* Accessed March, 2020. **10**: p. 2020.

**ALCOHOL HARMONISATION SYNTAX (SPSS)**

1. **EAS**

**CODING FOR CURRENT ABSTAINERS:*

**0=No; 1=Yes**

COMPUTE Current=0.

IF (SAB21=0 AND QtytoUse=0) Current=1.

IF (SAB21=1 AND QtytoUse=0 AND ((NOT SYSMIS(SAB22A)) OR (NOT SYSMIS(SAB23A)) OR (NOT SYSMIS(SAB24A)) OR (NOT SYSMIS(SAB25A)))) Current=1.

IF (SYSMIS(SAB21) AND QtytoUse=0) Current=88.

IF (SAB21=9 AND QtytoUse=0) Current=88.

IF (SAB21=20 AND QtytoUse=0) Current=1.

VARIABLE LABELS Current "Current abstinence".

VARIABLE LEVEL Current(NOMINAL).

Value labels Current 0 'Current drinker' 1 'Current abstainer'.

VARIABLE WIDTH Current(8).

EXECUTE.

RECODE Current (88=SYSMIS).

EXECUTE.

**CODING FOR LIFETIME ABSTAINERS:*

**0=No; 1=Yes**

Compute Lifetime1 = 0.

IF (SAB261.1=1 AND SAB262.1=1 AND SAB263.1 =1 AND SAB21.1=0) Lifetime1=1.

IF (SYSMIS(SAB261.1) OR SYSMIS(SAB262.1) OR SYSMIS(SAB263.1) OR SYSMIS (SAB21.1))

AND NOT

((SAB261.1 NE 1 AND NOT SYSMIS(SAB261.1)) OR (SAB262.1 NE 1 AND NOT SYSMIS(SAB262.1)) OR (SAB263.1 NE 1 AND NOT SYSMIS (SAB263.1)) OR (SAB21.1 NE 0 AND NOT SYSMIS(SAB21.1)))

Lifetime1=88.

value labels Lifetime1 0 'Has had alcohol' 1 'Lifetime abstainer'.

VARIABLE WIDTH Lifetime1(8).

EXECUTE.

RECODE Lifetime1 (88=SYSMIS).

EXECUTE.

Compute Lifetime2 = 0.

IF (Lifetime1=1 AND SAB263.2=1 AND SAB21.2=0) Lifetime2=1.

IF (SYSMIS(Lifetime1) AND SAB261.2=1 AND SAB262.2=1 AND SAB263.2=1 AND SAB21.2=0) Lifetime2=1.

IF (SYSMIS(Lifetime1) AND (SYSMIS(SAB261.2) OR SYSMIS(SAB262.2) OR SYSMIS(SAB263.2) OR SYSMIS(SAB21.2)))

AND NOT

((SAB261.2 NE 1 AND NOT SYSMIS(SAB261.2)) OR (SAB262.2 NE 1 AND NOT SYSMIS(SAB262.2)) OR (SAB263.2 NE 1 AND NOT SYSMIS(SAB263.2)) OR (SAB21.2 NE 0 AND NOT SYSMIS (SAB21.2)))

Lifetime2=88.

value labels Lifetime2 0 'Has had alcohol' 1 'Lifetime abstainer'.

VARIABLE WIDTH Lifetime2(8).

EXECUTE.

RECODE Lifetime2 (88=SYSMIS).

EXECUTE.

**NOTE: same coding scheme repeated across remaining waves.**

**CODING FOR GRAMS PER DAY:*

** First convert to AUS standard drinks -> 14g is a US standard drink; so multiply standard drinks by 1.4**

COMPUTE AUSdrinkspermonth=QtytoUSE*1.4.

IF (NOT SAB21=0) AND ((SYSMIS(SAB22A) OR SYSMIS(SAB22BN) OR SAB22B='0') AND (SYSMIS(SAB23A) OR SYSMIS(SAB23BN) OR SYSMIS(SAB24A) OR SYSMIS(SAB24BN) OR SYSMIS(SAB25A) OR SYSMIS(SAB25BN))) AUSdrinkspermonth=88.

RECODE AUSdrinkspermonth (88=SYSMIS).

EXECUTE.

COMPUTE DrinksDay=AUSdrinkspermonth / 30.

IF (SYSMIS(AUSdrinkspermonth)) DrinksDay=88.

EXECUTE.

RECODE DrinksDay (88=SYSMIS).

EXECUTE.

**Adding 10.06 -> convert to grams per day (not per drinking day)**

COMPUTE Grams_Day=DrinksDay*10.

EXECUTE.

**CODING FOR ALCOHOL CATEGORIES:*

** 0=lifetime abstainer; 1=current abstainer; 2= occasional; 3=low volume; 4; medium volume; 5=high volume; 6=higher volume**

COMPUTE DrinkCategory.1=22.

IF (Lifetime1=1) DrinkCategory.1=0.

IF ((Current.1=1) AND (Lifetime1=0 OR SYSMIS(Lifetime1))) DrinkCategory.1=1.

IF ((Current.1=0 OR SYSMIS(Current.1)) AND SYSMIS(DrinksDay.1)) DrinkCategory.1=88.

IF (SYSMIS(Current.1) AND (Lifetime1=0 OR SYSMIS(Lifetime1)) AND DrinksDay.1=0) DrinkCategory.1=1.

IF (Grams_Day.1 > 0 AND Grams_Day.1<1.3) DrinkCategory.1=2.

IF (Grams_Day.1>=1.3 AND Grams_Day.1<25) DrinkCategory.1=3.

IF (Grams_Day.1>=25 AND Grams_Day.1<45) DrinkCategory.1=4.

IF (Grams_Day.1>=45 AND Grams_Day.1<65) DrinkCategory.1=5.

IF (Grams_Day.1>=65) DrinkCategory.1=6.

IF (AFUN.1=1 AND SAB21.1=1 AND QtytoUSE.1=0) DrinkCategory.1=88.

Value labels DrinkCategory.1 0 'Lifetime Abstainer' 1 'Current Abstainer' 2 'Occasional' 3 'Low volume' 4 'Medium' 5 'High' 6 'Higher'.

VARIABLE WIDTH DrinkCategory.1(8).

EXECUTE.

RECODE DrinkCategory.1 (88=SYSMIS).

EXECUTE.

**Note: same coding scheme repeated across remaining waves**

1. **EPIDEMCA**

**Insufficient data to determine lifetime abstainers**

**CODING FOR CURRENT ABSTAINERS:*

COMPUTE Current.1=0.

IF (alcohol=0) Current.1=1.

IF (MISSING(alcohol)) Current.1=88.

IF ((MISSING(alcohol)) AND (unitbiere+unitlocal+unitalcohol=0)) Current.1=1.

IF (unitalcohol + unitlocal + unitbiere > 0) Current.1=0.

VARIABLE LABELS Current.1 "Current abstinence".

VARIABLE LEVEL Current.1(NOMINAL).

value labels Current.1 0 'Current drinker' 1 'Current abstainer'.

VARIABLE WIDTH Current.1(8).

EXECUTE.

RECODE Current.1 (88=SYSMIS).

EXECUTE.

**CODING FOR GRAMS PER DAY:*

COMPUTE Grams_Day.1=unitalcohol/ 7*10.

IF (MISSING(unitalcohol)) Grams_Day.1=88.

IF ((MISSING(unitalcohol)) AND NOT((MISSING(unitlocal)) AND (MISSING (unitbiere)))) Grams_Day.1=SUM(unitbiere to unitlocal).

EXECUTE.

RECODE Grams_Day.1 (88=SYSMIS).

EXECUTE.

**CODING FOR ALCOHOL CATEGORIES:*

** 0=lifetime abstainer; 1=current abstainer; 2= occasional; 3=low volume; 4; medium volume; 5=high volume; 6=higher volume**

COMPUTE DrinkCategory.1=22.

IF (Current.1=1) DrinkCategory.1=1.

IF (MISSING(Current.1)) DrinkCategory.1=88.

IF ((alcohol=1 OR alcohol=2) AND (((MISSING(unitlocal)) AND (MISSING(unitbiere)) AND (MISSING(unitalcohol))) OR

(SUM(unitbiere to unitalcohol) =0))) DrinkCategory.1=88.

IF (Grams_Day.1 > 0 AND Grams_Day.1<1.3) DrinkCategory.1=2.

IF (Grams_Day.1>=1.3 AND Grams_Day.1<25) DrinkCategory.1=3.

IF (Grams_Day.1>=25 AND Grams_Day.1<45) DrinkCategory.1=4.

IF (Grams_Day.1>=45 AND Grams_Day.1<65) DrinkCategory.1=5.

IF (Grams_Day.1>=65) DrinkCategory.1=6.

value labels DrinkCategory.1 1 'Current Abstainer' 2 'Occasional' 3 'Low volume' 4 'Medium' 5 'High' 6 'Higher'.

VARIABLE WIDTH DrinkCategory.1(8).

EXECUTE.

RECODE DrinkCategory.1 (88=SYSMIS).

EXECUTE.

**Note: coding for Current Abstainers, Grams Per Day, and Alcohol Categories repeated across remaining waves**

1. **ESPRIT**

**CODING FOR CURRENT ABSTAINERS:*

**0=No; 1=Yes**

**Long format data**

COMPUTE Current=0.

IF (BUVEUR=2 OR BUVEUR=3) Current=1.

IF (buveur2=0) Current=1.

IF (SYSMIS(BUVEUR) AND SYSMIS(buveur2)) Current=88.

VARIABLE LABELS Current "Current abstinence".

VARIABLE LEVEL Current(NOMINAL).

value labels Current 0 'Current drinker' 1 'Current abstainer'.

VARIABLE WIDTH Current(8).

EXECUTE.

RECODE Current (88=SYSMIS).

EXECUTE.

**CODING FOR LIFETIME ABSTAINERS:*

COMPUTE Lifetime.1=0.

IF (BUVEUR = 2) Lifetime.1=1.

IF SYSMIS(BUVEUR) Lifetime.1=88.

VARIABLE LABELS Lifetime.1 "Lifetime abstinence at baseline".

VARIABLE LEVEL Lifetime.1(NOMINAL).

value labels Lifetime.1 0 'Has had alcohol' 1 'Lifetime abstainer'.

VARIABLE WIDTH Lifetime.1(8).

EXECUTE.

RECODE Lifetime.1 (88=SYSMIS).

EXECUTE.

**CODING FOR GRAMS PER DAY:*

COMPUTE Grams_Day.1=ALCWEEK.0 / 7*10.

IF (SYSMIS(ALCWEEK.0)) Grams_Day.1=88.

EXECUTE.

RECODE Grams_Day.1 (88=SYSMIS).

EXECUTE.

**CODING FOR ALCOHOL CATEGORIES:*

** 0=lifetime abstainer; 1=current abstainer; 2= occasional; 3=low volume; 4; medium volume; 5=high volume; 6=higher volume**

COMPUTE DrinkCategory.1=22.

IF (Lifetime.1=1) DrinkCategory.1=0.

IF ((Current.0=1) AND (Lifetime.1=0 OR SYSMIS(Lifetime.1))) DrinkCategory.1=1.

IF ((Current.0=0 OR SYSMIS(Current.0)) AND SYSMIS(ALCWEEK.0)) DrinkCategory.1=88.

IF (SYSMIS(Current.0) AND (Lifetime.1=0 OR SYSMIS(Lifetime.1)) AND ALCWEEK.0=0) DrinkCategory.1=1.

IF (Grams_Day.1 > 0 AND Grams_Day.1<1.3) DrinkCategory.1=2.

IF (Grams_Day.1>=1.3 AND Grams_Day.1<25) DrinkCategory.1=3.

IF (Grams_Day.1>=25 AND Grams_Day.1<45) DrinkCategory.1=4.

IF (Grams_Day.1>=45 AND Grams_Day.1<65) DrinkCategory.1=5.

IF (Grams_Day.1>=65) DrinkCategory.1=6.

value labels DrinkCategory.1 0 'Lifetime Abstainer' 1 'Current Abstainer' 2 'Occasional' 3 'Low volume' 4 'Medium' 5 'High' 6 'Higher'.

VARIABLE WIDTH DrinkCategory.1(8).

EXECUTE.

RECODE DrinkCategory.1 (88=SYSMIS).

EXECUTE.

**Note: coding for Current Abstainers, Grams Per Day, and Alcohol Categories repeated across remaining waves**

1. **Framingham**

**Insufficient data to calculate lifetime abstainers**

**CODING FOR GRAMS PER DAY:*

Compute drinksdayg.1 = ((FH115 + FH116 + FH117)*14) / 7.

execute.

**CODING FOR ALCOHOL CATEGORIES:*

**Can't have lt 1 drink/week due to way questions asked so occasional drinkers coded as those having no more than 1 drink/week.*

** 0=lifetime abstainer; 1=current abstainer; 2= occasional; 3=low volume; 4; medium volume; 5=high volume; 6=higher volume**

do if (drinksdayg.1 = 0).

compute AlcCat.1 = 1.

else if (DrinksDayG.1 le 2).

compute AlcCat.1 = 2.

else if (DrinksDayG.1 ge 2) and (DrinksDayG.1 le 24.9).

compute AlcCat.1 = 3.

else if (DrinksDayG.1 ge 25) and (DrinksDayG.1 le 44.9).

compute AlcCat.1 = 4.

else if (DrinksDayG.1 ge 45) and (DrinksDayG.1 le 64.9).

compute AlcCat.1 = 5.

else if DrinksDayG.1 ge 65.

compute AlcCat.1 = 6.

end if.

execute.

**Note: same coding scheme repeated across remaining waves.**

1. **H70**

**CODING FOR LIFETIME ABSTAINERS:*

COMPUTE Lifetime1=0.

IF (PE1185=7) Lifetime1=1.

IF SYSMIS(PE1185) Lifetime1=88.

IF SYSMIS(PE1185) AND (PG1107=7) Lifetime1=1.

IF SYSMIS(PE1185) AND SYSMIS(PG1107) AND (AH397=7) Lifetime1=1.

VARIABLE LABELS Lifetime1 "Lifetime abstinence wave 1".

VARIABLE LEVEL Lifetime1(NOMINAL).

value labels Lifetime1 0 'Has had alcohol' 1 'Lifetime abstainer'.

VARIABLE WIDTH Lifetime1(8).

EXECUTE.

RECODE Lifetime1 (88=SYSMIS).

EXECUTE.

**CODING FOR CURRENT ABSTAINERS:*

**0 = current drinker 1= current abstainer*

**Note: testvar=1 means that none of the current frequency variables indicate drinking AND the vol variable does not indicate drinking either*

COMPUTE Current1=0.

IF ((PE1185=1 OR PE1185=2 OR PE1185=3 OR PE1185=4 OR PE1185=5 OR PE1185=6 OR PE1185=7) AND Testvar1=1) Current1=1.

IF Testvar1=1 AND NOT(MISSING(PE1186) AND MISSING(PE1189) AND MISSING(PE1193) AND MISSING(PE1196)) Current1=1.

IF (Lifetime1=1) Current1=1.

IF Testvar1=1 AND (MISSING(PE1186) AND MISSING(PE1189) AND MISSING(PE1193) AND MISSING(PE1196)) Current1=88.

IF (PE1185=0) Current1=0.

VARIABLE LABELS Current1 "Current abstinence".

VARIABLE LEVEL Current1(NOMINAL).

value labels Current1 0 'Current drinker' 1 'Current abstainer'.

VARIABLE WIDTH Current1(8).

EXECUTE.

RECODE Current1 (88=SYSMIS).

EXECUTE.

**CODING FOR GRAMS PER DAY:*

**Need to recode g/week categories into single numbers (take midpoints)**

**No country conversion needed as already measured in grams**

RECODE PE1196 (0=0)(1=10)(2=30)(3=50)(4=80)(5=125)(6=200)(7=375)(8=687.50) INTO PE1196_R.

EXECUTE.

COMPUTE Grams_Day.1 = PE1196_R/7.

EXECUTE.

**CODING FOR ALCOHOL CATEGORIES:*

** 0=lifetime abstainer; 1=current abstainer; 2= occasional; 3=low volume; 4; medium volume; 5=high volume; 6=higher volume*

COMPUTE DrinkCategory.1=22.

IF (Lifetime1=1) DrinkCategory.1=0.

IF ((Current1=1) AND (Lifetime1=0 OR SYSMIS(Lifetime1))) DrinkCategory.1=1.

IF ((Current1=0 OR SYSMIS(Current1)) AND SYSMIS(Grams_Day.1)) DrinkCategory.1=88.

IF ((Current1=0) AND (Grams_Day.1=0)) DrinkCategory.1=88.

IF (Grams_Day.1 > 0 AND Grams_Day.1<1.3) DrinkCategory.1=2.

IF (Grams_Day.1>=1.3 AND Grams_Day.1<25) DrinkCategory.1=3.

IF (Grams_Day.1>=25 AND Grams_Day.1<45) DrinkCategory.1=4.

IF (Grams_Day.1>=45 AND Grams_Day.1<65) DrinkCategory.1=5.

IF (Grams_Day.1>=65) DrinkCategory.1=6.

value labels DrinkCategory.1 0 'Lifetime Abstainer' 1 'Current Abstainer' 2 'Occasional' 3 'Low volume' 4 'Medium' 5 'High' 6 'Higher'.

VARIABLE WIDTH DrinkCategory.1(8).

EXECUTE.

RECODE DrinkCategory.1 (88=SYSMIS).

EXECUTE.

**Note: same coding scheme repeated across remaining waves**

1. **HELIAD**

**CODING FOR CURRENT ABSTAINERS:*

COMPUTE Current.1=0.

IF (C33=0) Current.1=1.

IF (MISSING(C33)) Current.1=88.

VARIABLE LABELS Current.1 "Current abstinence".

VARIABLE LEVEL Current.1(NOMINAL).

value labels Current.1 0 'Current drinker' 1 'Current abstainer'.

VARIABLE WIDTH Current.1(8).

EXECUTE.

RECODE Current.1 (88=SYSMIS).

EXECUTE.

**CODING FOR LIFETIME ABSTAINERS:*

**0=No 1=Yes***

**'9s' '3s' and '2s' and ‘999s’ = missing**

COMPUTE Lifetime.1=22.

IF ((C39 = 0) AND (Current.1=1 OR MISSING(Current.1))) Lifetime.1=1.

IF ((MISSING(C39)) OR (C39=9) OR (C39=3) OR (C39=2)) AND (Current.1=1 OR (MISSING(Current.1))) Lifetime.1=88.

IF (C33=1 OR C39=1 OR C41a>0 OR C42a>0 OR C43a>0 OR C44a>0) Lifetime.1=0.

VARIABLE LABELS Lifetime.1 "Lifetime abstinence".

VARIABLE LEVEL Lifetime.1(NOMINAL).

value labels Lifetime.1 0 'Has had alcohol' 1 'Lifetime abstainer'.

VARIABLE WIDTH Lifetime.1(8).

EXECUTE.

RECODE Lifetime.1 (88=SYSMIS).

EXECUTE.

**CODING FOR GRAMS PER DAY:*

**First converting frequency variable categories to days per week; taking midpoints of categories**

RECODE C41b (0=12)(1=6)(2=3.5)(3=.75)(4=.12)(5=.02) INTO C41b_R.

EXECUTE.

RECODE C42b (0=12)(1=6)(2=3.5)(3=.75)(4=.12)(5=.02) INTO C42b_R.

EXECUTE.

RECODE C43b (0=12)(1=6)(2=3.5)(3=.75)(4=.12)(5=.02) INTO C43b_R.

EXECUTE.

RECODE C44b (0=12)(1=6)(2=3.5)(3=.75)(4=.12)(5=.02) INTO C44b_R.

EXECUTE.

**Creating a number of drinks per week var - this will include historical volumes for people who used to drink but now no longer do**

COMPUTE TsipPerWeek = C41b_R*C41a.

EXECUTE.

COMPUTE BeerPerWeek = C42b_R*C42a.

EXECUTE.

COMPUTE WinePerWeek = C43b_R*C43a.

EXECUTE.

COMPUTE WhiskPerWeek = C44b_R*C44a.

EXECUTE.

COMPUTE DrinksPerWeek= SUM(TsipPerWeek, BeerPerWeek, WinePerWeek, WhiskPerWeek).

EXECUTE.

**Then convert drinks into AUS standard drinks**

**Using 13g for a Greek standard drink**

**Then convert AUS drinks per week into grams per day by multiplying by 10 and dividing by 7**

**Then convert into grams per day*

COMPUTE DrinksPerWeekAus = DrinksPerWeek * 1.3.

EXECUTE.

COMPUTE Grams_Day.1 = (DrinksPerWeekAus*10)/7.

EXECUTE.

**CODING FOR ALCOHOL CATEGORIES:*

** 0=lifetime abstainer; 1=current abstainer; 2= occasional; 3=low volume; 4; medium volume; 5=high volume; 6=higher volume*

COMPUTE DrinkCategory.1=22.

IF ((Current.1=0 OR MISSING(Current.1)) AND MISSING(Grams_Day.1)) DrinkCategory.1=88.

IF ((Current.1=0) AND (Grams_Day.1=0)) DrinkCategory.1=88.

IF (Grams_Day.1 > 0 AND Grams_Day.1<1.3) DrinkCategory.1=2.

IF (Grams_Day.1>=1.3 AND Grams_Day.1<25) DrinkCategory.1=3.

IF (Grams_Day.1>=25 AND Grams_Day.1<45) DrinkCategory.1=4.

IF (Grams_Day.1>=45 AND Grams_Day.1<65) DrinkCategory.1=5.

IF (Grams_Day.1>=65) DrinkCategory.1=6.

IF ((Current.1=0) AND (vnotf.1=1) AND (Grams_Day.1<65)) DrinkCategory.1=88.

**Put code for abstainers at the end to override historical alcohol consumption causing Grams_Day.1 variable to be >0 for abstainers*

IF (Lifetime.1=1) DrinkCategory.1=0.

IF ((Current.1=1) AND (Lifetime.1=0 OR MISSING(Lifetime.1))) DrinkCategory.1=1.

value labels DrinkCategory.1 0 'Lifetime Abstainer' 1 'Current Abstainer' 2 'Occasional' 3 'Low volume' 4 'Medium' 5 'High' 6 'Higher'.

VARIABLE WIDTH DrinkCategory.1(8).

EXECUTE.

RECODE DrinkCategory.1 (88=SYSMIS).

EXECUTE.

1. **KLOSCAD**

**CODING FOR CURRENT ABSTAINERS:*

**0=No 1=Yes**

**Long format data**

COMPUTE Current=0.

IF (AlcoholicDrinksPerWeek=0) Current=1.

IF (MISSING(AlcoholicDrinksPerWeek)) Current=88.

VARIABLE LABELS Current "Current abstinence".

VARIABLE LEVEL Current(NOMINAL).

value labels Current 0 'Current drinker' 1 'Current abstainer'.

VARIABLE WIDTH Current(8).

EXECUTE.

RECODE Current (88=SYSMIS).

EXECUTE.

**CODING FOR LIFETIME ABSTAINERS:*

**Lifetime abstainer 0=No 1=Yes**

**Wide format**

COMPUTE Lifetime.1=22.

IF (Lifetimealcoholconsumptionperweek.1=0) Lifetime.1=1.

IF MISSING(Lifetimealcoholconsumptionperweek.1) Lifetime.1=88.

IF (Lifetimealcoholconsumptionperweek.1>0 OR AlcoholicDrinksPerWeek.1>0 OR durationlifetimeconsumption.1>0 OR agebeginningofalcoholconsumption.1>0 OR ageabstainalcohol.1>0) Lifetime.1=0.

RECODE Lifetime.1 (88=SYSMIS).

EXECUTE.

COMPUTE Lifetime.2=22.

IF ((Lifetime.1=1) AND (Lifetimealcoholconsumptionperweek.2=0)) Lifetime.2=1.

IF (MISSING (Lifetime.1) AND Lifetimealcoholconsumptionperweek.2=0) Lifetime.2=1.

IF MISSING(Lifetimealcoholconsumptionperweek.2) Lifetime.2=88.

IF (Lifetime.1=0 OR Lifetimealcoholconsumptionperweek.2>0 OR AlcoholicDrinksPerWeek.2>0 OR durationlifetimeconsumption.2>0 OR agebeginningofalcoholconsumption.2>0 OR ageabstainalcohol.2>0) Lifetime.2=0.

RECODE Lifetime.2 (88=SYSMIS).

EXECUTE.

**Note: coding scheme for Lifetime Abstention repeated across remaining waves.**

**CODING FOR GRAMS PER DAY:*

**First convert drinks into AUS standard drinks**

**Using 11g for a Korean standard drink (average of the range found (8-14g)**

COMPUTE AlcoholicDrinksPerWeek.1_R = AlcoholicDrinksPerWeek.1 * 1.1.

EXECUTE.

COMPUTE Grams_Day.1=(AlcoholicDrinksPerWeek.1_R*10)/7.

EXECUTE.

**Note: coding scheme for Grams Per Day repeated across remaining waves.**

**CODING FOR ALCOHOL CATEGORIES:*

**0=lifetime abstainer; 1=current abstainer; 2= occasional; 3=low volume; 4; medium volume; 5=high volume; 6=higher volume**

COMPUTE DrinkCategory.1=22.

IF (SYSMIS(Current.1)) DrinkCategory.1=88.

IF (Lifetime.1=1) DrinkCategory.1=0.

IF ((Current.1=1) AND (Lifetime.1=0 OR SYSMIS(Lifetime.1))) DrinkCategory.1=1.

IF (Grams_Day.1 > 0 AND Grams_Day.1<1.3) DrinkCategory.1=2.

IF (Grams_Day.1>=1.3 AND Grams_Day.1<25) DrinkCategory.1=3.

IF (Grams_Day.1>=25 AND Grams_Day.1<45) DrinkCategory.1=4.

IF (Grams_Day.1>=45 AND Grams_Day.1<65) DrinkCategory.1=5.

IF (Grams_Day.1>=65) DrinkCategory.1=6.

value labels DrinkCategory.1 0 'Lifetime Abstainer' 1 'Current Abstainer' 2 'Occasional' 3 'Low volume' 4 'Medium' 5 'High' 6 'Higher'.

VARIABLE WIDTH DrinkCategory.1(8).

EXECUTE.

RECODE DrinkCategory.1 (88=SYSMIS).

EXECUTE.

**Note: coding scheme for Alcohol Categories repeated across remaining waves.**

1. **LEILA**

**CODING FOR GRAMS PER DAY:*

**Need to recode less than monthly as 0.1, otherwise everything else is coded consistently with other studies.*

RECODE Alcohol1_Frequency (0=0.1) (else=copy) into freq.1.

EXECUTE.

compute DrinksDayG.1 = ((Alcohol1_Drinks_per_Day*freq.1)*11)/7.

execute.

**CODING FOR ALCOHOL CATEGORIES:*

** 0=lifetime abstainer; 1=current abstainer; 2= occasional; 3=low volume; 4; medium volume; 5=high volume; 6=higher volume*

do if DrinksDayG.1 = 0.

compute AlcCat.1 = 1.

else if (DrinksDayG.1 lt 1.3).

compute AlcCat.1 = 2.

else if (DrinksDayG.1 ge 1.3) and (DrinksDayG.1 le 24.9).

compute AlcCat.1 = 3.

else if (DrinksDayG.1 ge 25) and (DrinksDayG.1 le 44.9).

compute AlcCat.1 = 4.

else if (DrinksDayG.1 ge 45) and (DrinksDayG.1 le 64.9).

compute AlcCat.1 = 5.

else if DrinksDayG.1 ge 65.

compute AlcCat.1 = 6.

end if.

execute.

do if alcohol1_ever = 0.

recode alccat.1 (1=0).

end if.

execute.

**Note: same coding scheme repeated across remaining waves**

1. **MAAS**

**Insufficient data to calculate lifetime abstainers**

**CODING FOR GRAMS PER DAY:*

do if alcohol_numeric.0 = 1.

recode alcday_numeric.0 (-1=0).

end if.

execute.

recode alcday_numeric.0 (-1=sysmis).

execute.

do if alcohol_numeric.0 = 1.

compute alcweek_numeric.0 =0.

end if.

execute.

recode alcweek_numeric.0 (6=sysmis).

execute.

recode alcday_numeric.0 (0=0)(1=1.5)(2=4.5)(3=8.5)(4=10.75) into quant.1.

execute.

recode alcweek_numeric.0 (0=0) (1=0.5)(2=1.5)(3=3.5)(4=5.5)(5=7) into freq.1.

execute.

compute Grams_Day.1 = freq.1*quant.1/7*10.

execute.

**CODING FOR ALCOHOL CATEGORIES:*

** 0=lifetime abstainer; 1=current abstainer; 2= occasional; 3=low volume; 4; medium volume; 5=high volume; 6=higher volume*

do if Grams_Day.1 = 0.

compute AlcCat.1 = 1.

else if (Grams_Day.1 lt 1.3).

compute AlcCat.1 = 2.

else if (Grams_Day.1 ge 1.3) and (Grams_Day.1 le 24.9).

compute AlcCat.1 = 3.

else if (Grams_Day.1 ge 25) and (Grams_Day.1 le 44.9).

compute AlcCat.1 = 4.

else if (Grams_Day.1 ge 45) and (Grams_Day.1 le 64.9).

compute AlcCat.1 = 5.

else if Grams_Day.1 ge 65.

compute AlcCat.1 = 6.

end if.

execute.

**Note: coding for Grams Per Day and Alcohol Categories repeated across remaining waves.**

1. **MYHAT**

**CODING FOR LIFETIME ABSTAINERS:*

COMPUTE Lifetime.1=0.

IF (ever_drink=0) Lifetime.1=1.

IF MISSING(ever_drink) Lifetime.1=88.

RECODE Lifetime.1 (88=SYSMIS).

EXECUTE.

COMPUTE Lifetime.2=0.

IF (Lifetime.1=1 AND drink2=0) Lifetime.2=1.

IF (MISSING(Lifetime.1) OR MISSING(drink2)) Lifetime.2=88.

RECODE Lifetime.2 (88=SYSMIS).

EXECUTE.

**Note: coding scheme for Lifetime Abstainers repeated across remaining waves.**

**CODING FOR CURRENT ABSTAINERS:*

**0=No 1=Yes**

COMPUTE Current.1=0.

IF (ever_drink=0 OR ever_drink=1) Current.1=1.

IF MISSING(ever_drink) Current.1=88.

RECODE Current.1 (88=SYSMIS).

EXECUTE.

COMPUTE Current.2=0.

IF (drink2=0) Current.2=1.

IF MISSING(drink2) Current.2=88.

RECODE Current.2 (88=SYSMIS).

EXECUTE.

**Note: coding scheme for Current Abstainers repeated across remaining waves.**

**CODING FOR GRAMS PER DAY:*

**First convert to midpoint of volume categories, then convert to Australian standard drinks**

**-2 is the study code for has not drunk in the past year**

RECODE drinkamt1 (-2=88)(1=1)(2=2.5)(3=4.5)(4=6.75) INTO quant.1.

EXECUTE.

RECODE quant.1 (88=SYSMIS).

EXECUTE.

**convert to mid-point for frequency as well**

recode drinkfreq1 (-2=88)(1=7)(2=4)(3=1)(4=0.75)(5=0.25) into freq.1.

execute.

recode freq.1 (88=SYSMIS).

execute.

compute drinksdayg.1 = freq.1*quant.1/7*14.

execute.

**CODING FOR ALCOHOL CATEGORIES:*

** 0=lifetime abstainer; 1=current abstainer; 2= occasional; 3=low volume; 4; medium volume; 5=high volume; 6=higher volume*

**Calculated using both volume and frequency data**

do if lifetime.1 = 1.

compute AlcCat.1 = 0.

else if current.1 = 1.

compute AlcCat.1 = 1.

else if (DrinksDayG.1 lt 1.3).

compute AlcCat.1 = 2.

else if (DrinksDayG.1 ge 1.3) and (DrinksDayG.1 le 24.9).

compute AlcCat.1 = 3.

else if (DrinksDayG.1 ge 25) and (DrinksDayG.1 le 44.9).

compute AlcCat.1 = 4.

else if (DrinksDayG.1 ge 45) and (DrinksDayG.1 le 64.9).

compute AlcCat.1 = 5.

else if DrinksDayG.1 ge 65.

compute AlcCat.1 = 6.

end if.

execute.

**Note: coding scheme used for Grams Per Day and Alcohol Categories repeated across remaining waves.**

1. **PATH**

**CODING FOR GRAMS PER DAY:*

**First need to recode AUDIT ranges into mid-points.*

recode audit1s (1=0)(2=0)(3=0.25)(4=0.75)(5=2.5)(6=5.5) into freq.1.

execute.

recode audit2 (1=1.5)(2=3.5)(3=5.5)(4=8)(5=10.75) into quant.1.

execute.

compute drinksdayg.1 = freq.1*quant.1/7*10.

execute.

**CODING FOR ALCOHOL CATEGORIES:*

** 0=lifetime abstainer; 1=current abstainer; 2= occasional; 3=low volume; 4; medium volume; 5=high volume; 6=higher volume*

do if audit1s = 1.

compute AlcCat.1 = 0.

else if (drinksdayg.1 = 0).

compute AlcCat.1 = 1.

else if (DrinksDayG.1 lt 1.3).

compute AlcCat.1 = 2.

else if (DrinksDayG.1 ge 1.3) and (DrinksDayG.1 le 24.9).

compute AlcCat.1 = 3.

else if (DrinksDayG.1 ge 25) and (DrinksDayG.1 le 44.9).

compute AlcCat.1 = 4.

else if (DrinksDayG.1 ge 45) and (DrinksDayG.1 le 64.9).

compute AlcCat.1 = 5.

else if DrinksDayG.1 ge 65.

compute AlcCat.1 = 6.

end if.

execute.

**Note: coding for Grams Per Day and Alcohol Categories repeated for remaining waves.**

1. **SALSA**

**Insufficient data to calculate lifetime abstainers**

**CODING FOR GRAMS PER DAY:*

COMPUTE DrinksDayG.1 = BL_alc*14/7.

EXECUTE.

**CODING FOR CURRENT ABSTAINER:*

DO IF MH65 = 2 AND MH66 = 2 AND MH67 = 2.

COMPUTE Drinker.1 = 0.

ELSE IF MH65 = 1 OR MH66 = 1 OR MH67 = 1.

COMPUTE Drinker.1 = 1.

END IF.

EXECUTE.

**CODING FOR ALCOHOL CATEGORIES:*

** 0=lifetime abstainer; 1=current abstainer; 2= occasional; 3=low volume; 4; medium volume; 5=high volume; 6=higher volume*

**Create categories without lifetime abstainer (AlcCat = 0).*

do if Drinker.1 = 0.

compute AlcCat.1 = 1.

else if (DrinksDayG.1 lt 1.3).

compute AlcCat.1 = 2.

else if (DrinksDayG.1 ge 1.3) and (DrinksDayG.1 le 24.9).

compute AlcCat.1 = 3.

else if (DrinksDayG.1 ge 25) and (DrinksDayG.1 le 44.9).

compute AlcCat.1 = 4.

else if (DrinksDayG.1 ge 45) and (DrinksDayG.1 le 64.9).

compute AlcCat.1 = 5.

else if DrinksDayG.1 ge 65.

compute AlcCat.1 = 6.

end if.

execute.

**Note: same coding scheme repeated across remaining waves.**

1. **SPAH**

**CODING FOR GRAMS PER DAY:*

**Standard drink in Brazil is 12g.*

compute DrinksDayG.1 = palcnow*12/7.

execute.

**CODING FOR ALCOHOL CATEGORIES:*

** 0=lifetime abstainer; 1=current abstainer; 2= occasional; 3=low volume; 4; medium volume; 5=high volume; 6=higher volume*

do if DrinksDayG.1 = 0.

compute AlcCat.1 = 1.

else if (DrinksDayG.1 lt 1.3).

compute AlcCat.1 = 2.

else if (DrinksDayG.1 ge 1.3) and (DrinksDayG.1 le 24.9).

compute AlcCat.1 = 3.

else if (DrinksDayG.1 ge 25) and (DrinksDayG.1 le 44.9).

compute AlcCat.1 = 4.

else if (DrinksDayG.1 ge 45) and (DrinksDayG.1 le 64.9).

compute AlcCat.1 = 5.

else if DrinksDayG.1 ge 65.

compute AlcCat.1 = 6.

end if.

execute.

**Recode lifetime abstainers to 0.*

do if palcpast = 0 and drinksdayg.1 = 0.

recode alccat.1 (1=0).

end if.

execute.

1. **Sydney MAS**

**CODING FOR GRAMS PER DAY:*

**Frequency data in ranges so take mid-points. 1 = not in last year, 2 = less than monthly, 3 = 3 times/month, 4 = 2.5 times/week, 5 = 5 times/week, 6 = 7 times/week*

**Quantity data in ranges so need to take mid-points. 1 = 1, 2 = 2.5, 3 = 4.5, 4 = 6.5, 5 = 8.75*

**Occasional = 2 or 3 freq and 1 or 2 quant; Moderate = 4+ freq and 1 or 2 quant; Heavy = 3+ quant.*

**0=lifetime abstainer; 1=current abstainer; 2= occasional; 3 = moderate ; 4=heavy.*

recode etoh_freq (1=0)(2=0.25)(3=0.75)(4=2.5)(5=5)(6=7) into freq.1.

execute.

recode etoh_amount (1=1)(2=2.5)(3=4.5)(4=6.5)(5=8.75) into quant.1.

execute.

compute drinksdayg.1 = freq.1*quant.1/7*10.

execute.

**CODING FOR ALCOHOL CATEGORIES:*

** 0=lifetime abstainer; 1=current abstainer; 2= occasional; 3=low volume; 4; medium volume; 5=high volume; 6=higher volume*

do if (drinksdayg.1 = 0).

compute AlcCat.1 = 1.

else if (DrinksDayG.1 lt 1.3).

compute AlcCat.1 = 2.

else if (DrinksDayG.1 ge 1.3) and (DrinksDayG.1 le 24.9).

compute AlcCat.1 = 3.

else if (DrinksDayG.1 ge 25) and (DrinksDayG.1 le 44.9).

compute AlcCat.1 = 4.

else if (DrinksDayG.1 ge 45) and (DrinksDayG.1 le 64.9).

compute AlcCat.1 = 5.

else if DrinksDayG.1 ge 65.

compute AlcCat.1 = 6.

end if.

execute.

**Recode so abstainers are split into current and lifetime.*

DO IF (etoh_ever = 1).

RECODE AlcCat.1 (1=0).

END IF.

EXECUTE.

**Note: coding for Grams Per Day and Alcohol Categories repeated across remaining waves**

1. **ZARADEMP**

**CODING FOR GRAMS PER DAY:*

**Compute weekly number of wine drinks from weekday and weekend consumption.*

COMPUTE WineWD.1=A05102 * 5.

VARIABLE LABELS WineWD.1 'Weekday wine consumption'.

EXECUTE.

COMPUTE WineWE.1=A05103 * 2.

VARIABLE LABELS WineWE.1 'Weekend wine consumption'.

EXECUTE.

COMPUTE Wine.1 = WineWD.1 + WineWE.1.

VARIABLE LABELS Wine.1 'Weekly wine SD'.

EXECUTE.

**Compute weekly number of Beer drinks from weekday and weekend consumption.*

COMPUTE BeerWD.1=A05108 * 5.

VARIABLE LABELS BeerWD.1 'Weekday Beer consumption'.

EXECUTE.

COMPUTE BeerWE.1=A05109 * 2.

VARIABLE LABELS BeerWE.1 'Weekend Beer consumption'.

EXECUTE.

COMPUTE Beer.1 = BeerWD.1 + BeerWE.1.

VARIABLE LABELS Beer.1 'Weekly Beer SD'.

EXECUTE.

**Compute weekly number of Spirit drinks from weekday and weekend consumption.*

COMPUTE SpiritWD.1=A05114 * 5.

VARIABLE LABELS SpiritWD.1 'Weekday Spirit consumption'.

EXECUTE.

COMPUTE SpiritWE.1=A05115 * 2.

VARIABLE LABELS SpiritWE.1 'Weekend Spirit consumption'.

EXECUTE.

COMPUTE Spirit.1 = (SpiritWD.1 + SpiritWE.1)*2.

VARIABLE LABELS Spirit.1 'Weekly Spirit SD'.

EXECUTE.

**Recode those who are not never drinkers but have a missing code for wine into 0 wine drinks on weekdays and weekends.*

DO IF (a05101r NE 0).

RECODE Wine.1 (SYSMIS=0).

END IF.

EXECUTE.

**Recode those who are not never drinkers but have a missing code for beer into 0 beer drinks on weekdays and weekends.*

DO IF (a05101r NE 0).

RECODE Beer.1 (SYSMIS=0).

END IF.

EXECUTE.

**Recode those who are not never drinkers but have a missing code for spirit into 0 spirits drinks on weekdays and weekends.*

DO IF (a05101r NE 0).

RECODE Spirit.1 (SYSMIS=0).

END IF.

EXECUTE.

COMPUTE DrinksWeek.1 = Wine.1 + Beer.1 + Spirit.1.

VARIABLE LABELS DrinksWeek.1 'Number of standard drinks per week'.

EXECUTE.

COMPUTE DrinksDayG.1 = (DrinksWeek.1/7)*10.

VARIABLE LABELS DrinksDayG.1 'Number of grams per day'.

EXECUTE.

**CODING FOR ALCOHOL CATEGORIES*

** 0=lifetime abstainer; 1=current abstainer; 2= occasional; 3=low volume; 4; medium volume; 5=high volume; 6=higher volume*

do if a05101r = 0.

compute AlcCat.1 = 0.

else if a05101r = 3 and drinksdayg.1=0.

compute AlcCat.1 = 1.

else if (DrinksDayG.1 lt 1.3).

compute AlcCat.1 = 2.

else if (DrinksDayG.1 ge 1.3) and (DrinksDayG.1 le 24.9).

compute AlcCat.1 = 3.

else if (DrinksDayG.1 ge 25) and (DrinksDayG.1 le 44.9).

compute AlcCat.1 = 4.

else if (DrinksDayG.1 ge 45) and (DrinksDayG.1 le 64.9).

compute AlcCat.1 = 5.

else if DrinksDayG.1 ge 65.

compute AlcCat.1 = 6.

end if.

execute.

**Note: coding for Grams Per Day and Alcohol Categories repeated across remaining waves.**
